# Performance enhancement of electrochemical discharge micromachining of borosilicate glass using nitrogen gas assistance

**DOI:** 10.1038/s41598-026-36060-w

**Published:** 2026-02-12

**Authors:** Sekar Tamilperuvalathan, Vinoth Varadharaju, Sakthivel Rajamohan, Dhinesh Balasubramanian, Utku Kale, Artūras Kilikevičius

**Affiliations:** 1 Department of Mechanical Engineering, Government College of Technology, Coimbatore, India; 2 Department of Mechanical Engineering, Dhirajlal Gandhi College of Technology, Salem, India; 3 Department of Port Engineering, Lithuanian Maritime Academy (LMA) Vilnius Gediminas Technical University, Klaipėda, Lithuania; 4 Department of Aeronautics and Naval Architecture, Faculty of Transportation Engineering and Vehicle Engineering, Budapest University of Technology and Economics, Műegyetem rkp. 3., Budapest, H-1111 Hungary; 5 Mechanical Science Institute, Vilnius Gediminas Technical University, Plytinės g. 25, 10105 Vilnius, Lithuania

**Keywords:** ECDµM, Material removal, Tool wear, RSM, GRA, Random forest algorithm, Energy science and technology, Engineering, Materials science

## Abstract

Electrochemical Discharge Micro-Machining (ECDµM) of borosilicate glass commonly suffers from unstable discharges, surface cracking, low material removal (MR), and excessive tool wear (TW) when conventional liquid or air-based dielectrics are used. These issues limit machining efficiency and sustainable process development, especially when MR and TW are optimized independently. To overcome these limitations, this study investigates nitrogen gas as a regulated gaseous dielectric in combination with an aqueous sodium hydroxide (NaOH) electrolyte to enhance discharge stability and achieve balanced machining performance. Experiments were conducted by varying applied voltage, NaOH concentration and nitrogen gas flow rate, and the responses were analyzed using Response Surface Methodology (RSM), Grey Relational Analysis (GRA), and a Random Forest Algorithm (RFA). Stable machining was achieved within a nitrogen gas flow range of 3–5 L/min. At 120 V and 30 wt% NaOH, increasing the nitrogen gas flow rate from 3 to 5 L/min maintained MR at 4 mg while reducing TW from 3 to 2 mg. The optimal condition (134 V, 20 wt% NaOH, 4 L/min nitrogen gas flow rate) yielded 5 mg MR with negative TW (− 1 mg), while reducing electrolyte concentration by 33%. The Random Forest model predicted near-optimal parameters (119.89 V, 20.107 wt% NaOH, 4.31 L/min) with an average deviation of approximately 8%. These results establish nitrogen gas assisted ECDµM as a stable, energy-efficient and environmentally responsible approach for precision micromachining of brittle materials.

## Introduction

The demand for micro-scale glass components has increased significantly due to their role in miniaturized devices that require optical transparency, chemical inertness, high hardness, and stability under UV exposure. Borosilicate glass, in particular, is widely used in advanced industrial and medical applications because of its favourable physical and chemical properties. However, its brittleness and high hardness make it difficult to machine using both conventional and non-conventional processes, highlighting the need for alternative and innovative micromachining techniques^1^. Among these, borosilicate glass stands out for its exceptional physical and chemical characteristics, making it indispensable in advanced industrial applications and medical scientific reports technologies. However, its inherent brittleness and hardness present significant machining challenges, making both conventional and non-conventional machining methods difficult to implement. This underscores the need for innovative techniques to address these limitations in machining borosilicate glass^2,3^. Electrochemical Discharge Micro-Machining (ECDµM) has emerged as a promising unconventional technique for machining both conductive and non-conductive materials. While Electrochemical Machining (ECM) and Electric Discharge Machining (EDM) are widely used for micromachining borosilicate glass components in Micro-Electro-Mechanical Systems (MEMS) and Nano-Electro-Mechanical Systems (NEMS), ECDµM combines the advantages of these processes^4^. The main factors influencing material removal (MR) and tool wear (TW) in ECDµM include applied voltage, duty cycle, concentration of electrolytes, characteristics of gas film at the machining gap, electrical conductivity of the cathode materials and auxiliary anode electrode materials, the distance between the workpiece and auxiliary electrode, cathode electrode coatings, electrolyte temperature, and the surface area of the auxiliary anode. Among these, the formation and stability of the gas film play a critical role in determining ECDµM performance by enabling effective energy distribution in the machining zone, which enhances MR and reduces TW^5–8^. MR in ECDµM occurs through a combination of chemical erosion, localized evaporation, thermally induced chemical etching, and electrochemical reactions^9,10^. However, achieving optimal MR and TW simultaneously remains a challenge, as most studies focus on optimizing these objectives individually. In general, the term electrolyte is commonly associated with ECM, while dielectric medium is specific to EDM. In ECM, electrolytes such as NaOH, KOH, NaNO₃, NaCl, and polymer-based solutions are selected based on process requirements like precision, MR, Overcut (OC), and minimization of the Heat-Affected Zone (HAZ). Conversely, in EDM, dielectric media typically include kerosene, deionized water, and glycol–water mixtures for wet-type machining. Traditionally, kerosene oil is considered as a dielectric medium for a long time, even though it releases dangerous gases, vapors, and fumes that are hurtful to the environment and operator health. In related investigations on wire electric discharge machining of hardened AISI D2 and DC53 tool steels, Taguchi L18 orthogonal arrays together with analysis of variance and weighted signal-to-noise based multi-response optimization have been employed to control kerf width for flat, inclined, and curved profiles, as well as machining time, leading to substantial improvements in these performance measures^11^.

The recently published research articles related to various dielectric media used in EDM and its allied non-traditional machining processes, along with their potential challenges, are tabulated in Table 1.

**Table 1:** Various Dielectric media and their Challenges.

Dielectric medium/Prediction/Optimization models	Material	Challenges involved
EDM—Powder Al₂O₃ mixed with Deionized (DI) water Response Surface Methodology (RSM) + Grey Relational Analysis (GRA)^12^	Al6061	Maintaining conductivity and stable spark generation in DI water demands higher energy & Disposal of used electrolytes
EDM—Powder Al₂O₃ mixed with DI water Artificial Neural Network (ANN) + Non-Sorting Genetic Algorithm (NSGA-II)^13^		
EDM—Kerosene^14^	SS310	It releases dangerous gases, vapors, and fumes that are hurtful to the environment and operator health
EDM—Graphene mixed dielectric ANN + Multi-Objective Genetic Algorithm (MOGA)^15^	SS316	Waste dielectric containing graphene can pose disposal and contamination concerns
WEDM—DI water Mixed Level Taguchi + Analysis of Variance (ANOVA)^16^	AISI D2	Debris accumulation hinders spark initiation and energy transfer
EDM—Surfactants into the dielectric fluid ANN^17^	Inconel 617	Surfactants may lose effectiveness at elevated machining temperatures, reducing their ability to control debris dispersion
WEDM—DI Water Taguchi and GRA^18^	Ti6Al4V alloy	DI water causes high electrode wear in conventional EDM setups due to stagnant dielectric
WEDM—DI water ANOVA and RSM^19^	AISI D2/ DC53 tool steel	
High Speed WEDM—Resin (MBQR400) was combined with DI water^20^	HAMCs	Disposal of resin-contaminated DI water can pollute soil and water if not treated properly
EDM—Non-ionic liquid-mixed with kerosene Full factorial experiment^21^	Ti6Al4V	Disposal and biodegradability of non-ionic liquids are uncertain, raising ecological concerns
EDM—Alumina-mixed DI water^22^	Al6061	Continuous stirring or circulation is needed to keep alumina nanoparticles suspended, increasing energy demand
EDM—SiC Powder mixed with DI water GRA^23^	Ti6Al4V ELI	Waste DI water containing SiC nanoparticles is difficult to treat and may cause soil and water contamination. Maintaining uniform suspension of SiC powder requires continuous stirring, increasing energy demand
EDM—SiC Powder mixed with DI water Adaptive Neuro-Fuzzy Inference System (ANFIS)^24^		
EDM—Surfactant-mixed kerosene Statistical analysis^25^	Ti6Al4V	The low surfactant content in kerosene-based dielectrics leads to poor debris dispersion, resulting in excessive surface defects
WEDM—DI water including a de-ionizing agent zeolite ANOVA + TAGUCHI + GRA^26^	Inconel 718	Maintaining the conductivity of used dielectric and ability to maintain spark stabilization
EDM—Surfactant and aluminum powder in the dielectric-Kerosene RSM^27^	D2- Steel	Disposal of kerosene with surfactants and Al powder poses severe environmental hazards due to toxicity and non-biodegradability

For dry-type EDM, compressed air, argon, and other inert gases are employed. However, conventional dielectrics such as kerosene pose serious health and environmental hazards. Moreover, issues such as oxide formation during machining, high thermal dissipation to the surrounding, localized heat accumulation, and high ionization energy often contribute to an expanded heat-affected zone and the formation of micro-cracks—especially in brittle materials like glass.

Stainless steel is commonly used as a tool electrode in ECDµM due to its resistance to the corrosive nature of aqueous NaOH electrolytes, even though other materials such as tungsten carbide, tungsten, copper, high-carbon steel, and high-speed steel are available. Additionally, the surface characteristics of the auxiliary electrode significantly influence gas film formation by aiding hydrogen bubble coalescence between the tool electrode and workpiece^28^. Although ECDµM has shown promise for machining borosilicate glass and other difficult-to-cut materials, several critical challenges remain unresolved. In particular, the formation and stability of the gas film in the machining gap are not yet fully understood, which complicates the simultaneous control of MR and TW. Most existing studies employ conventional liquid dielectrics or dry media and typically optimize MR and TW separately, making it difficult to obtain a balanced, high-performance window for industrial applications^29,30^. The effects of applied voltage, duty ratio, pulse cycle time and concentration on material removal rate (MRR) and hole radial overcut (ROC) in borosilicate glass using Response Surface Methodology (RSM) were explored^31^. Taguchi method was employed to study MRR optimization while machining borosilicate glass using stainless steel tools and NaOH electrolyte^32^. The application of liquid nitrogen within a minimal-quantity lubrication approach during the machining of high-temperature-resistant alloys results in lower specific cutting energy demands, thereby supporting environmentally responsible manufacturing^33^. Development of the design of experiments using Taguchi methods for research activities have been demonstrated^34^. GRA was applied to optimize MRR while minimizing OC and taper in ECDµM process, and similar optimization approaches have been reported for improving performance characteristics in powder-mixed EDM of H-11 die steel^35,36^. GRA was applied to determine the optimal machining parameters for Nimonic 90, a critical material used in gas turbine components. Validation experiments confirmed the reliability of the optimization model, with less than 2% deviation between predicted and experimental results^37^. An experimental investigation was carried out to assess the machinability of a sustainable Al/SiC/Gr hybrid composite, with GRA employed to evaluate performance trends and to explain the underlying mechanisms responsible for the observed improvements^38^. Machine learning techniques are increasingly being used in machining optimization.

Recent advances in ECDµM have leveraged artificial intelligence models, such as decision trees and neural networks, to enhance process parameter optimization and machining outcomes. In parallel, alternative dielectric media—particularly nitrogen gas—have been explored to improve machining zone stability and surface characteristics by stabilizing the gas film and minimizing adverse thermal effects^39–43^. Despite these advancements, systematic studies addressing environmental concerns, energy efficiency, and sustainable manufacturing practices remain limited^44,45^. Prior work has primarily examined conventional dielectric media such as DI water and air, employing optimization methods that could be insufficient to capture complex nonlinear parameter interactions^46^. To mitigate these challenges, the present study utilizes aqueous NaOH, prepared using DI water at varying NaOH concentrations, as the electrolyte. Simultaneously, nitrogen gas is employed as the dielectric medium under different discharge parameters to evaluate its effectiveness in reducing thermal damage, improving MR, and enhancing machining quality. Recent studies have compared DI water and kerosene as EDM dielectric fluids, assessing performance in terms of MR, surface finish, and environmental impact, thus providing a reference for evaluating nitrogen-assisted machining. Moreover, sustainability-oriented reviews highlight the health, safety, and environmental implications of nitrogen, DI water, and kerosene, emphasizing nitrogen’s potential as a more eco-friendly and cost-effective alternative^47,48^.

Despite considerable work on ECDµM and related processes, there is still limited understanding of how dielectric media, particularly gaseous dielectrics, affect the simultaneous optimization of MR and TW under environmentally conscious conditions. Existing studies often treat MR and TW separately and rarely account for nonlinear interactions between process parameters. The present work addresses these gaps by investigating nitrogen gas as a dielectric medium in ECDµM of borosilicate glass, in combination with an aqueous NaOH electrolyte, and by applying RSM, GRA and RFA to model and optimize the process responses^49^.

However, several important gaps remain in the existing ECDµM and related non-traditional machining literature. First, most studies treat MR and TW as separate objectives and do not explicitly address their coupled behavior, making it difficult to identify operating windows that provide a balanced trade-off between productivity and tool life. Second, the role of gas-film formation and stability in the machining gap, particularly when gas is introduced in a controlled manner, is still not fully clarified, even though it has a critical influence on discharge behavior, thermal loading, and surface integrity in brittle glass substrates. Third, many reported optimization and modelling approaches rely predominantly on conventional statistical techniques, which may not adequately capture nonlinear interactions among process parameters such as applied voltage, electrolyte concentration, and gas flow rate, and thus offer limited predictive capability for process planning and sustainable operation.

The novelty of the present work is demonstrated through three key contributions. First, nitrogen gas is introduced as a regulated gaseous environment in the ECDµM of borosilicate glass, and its role in influencing gas-film stability, MR and TW is systematically examined in the presence of an aqueous NaOH electrolyte. Second, MR and TW are treated as interdependent performance responses, and GRA is employed to determine balanced operating conditions that enhance overall process performance, rather than optimizing individual responses in isolation as commonly reported in earlier studies. Third, a RFA–based predictive model is developed and validated to estimate MR and TW across varying applied voltage, electrolyte concentration and nitrogen flow rate, offering a data-driven approach for identifying stable and sustainable micromachining conditions for borosilicate glass.

## Methodology and experimentation

**Fig. 1:**
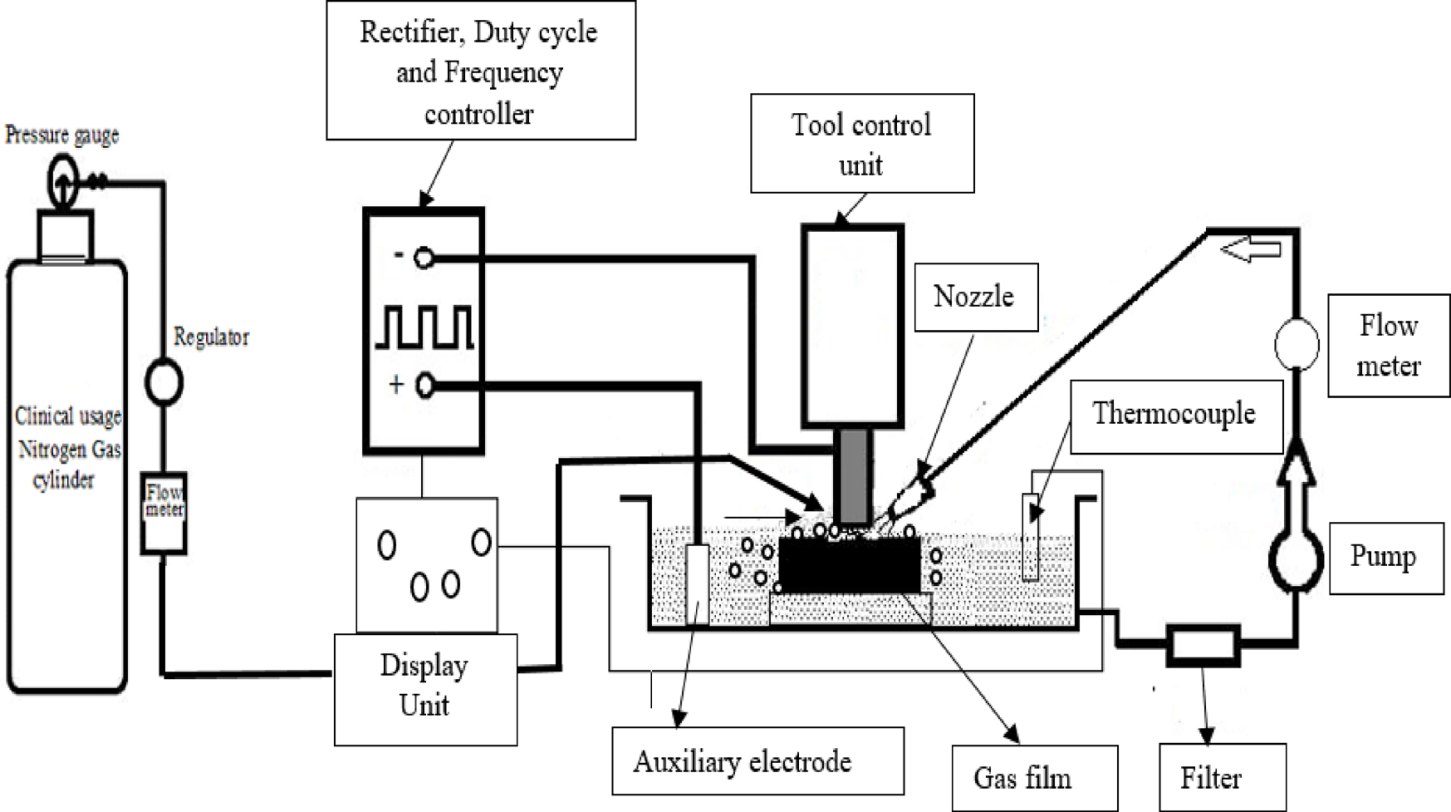
Schematic view of an indigenously developed ECDµM setup.

Figure 1 illustrates a fundamental schematic representation of the ECDµM process. The setup comprises a power source that supplies a DC voltage by pulsed forms between the cathode as a tool electrode and anode as an auxiliary electrode and a pulse generator for regulating the voltage^50^. A key distinction between ECDµM and ECM is that, in ECDµM, the workpiece is connected to the positive terminal (anode)^46^. Consequently, the electrochemical equivalence and electrical conductivity of the workpiece material play an essential role in the MR mechanism. However, unlike ECM, ECDµM does not depend on the electrical conductivity of the workpiece material during the machining process. Instead, it operates independently of the material’s conductive properties. The process involves the formation of bubbles near both electrodes due to electrolysis induced by the applied potential difference.

As the critical voltage increases, these bubbles coalesce and expand, forming a dense, cohesive gas film that initiates and sustains the MR mechanism^51,52^.

**Fig. 2:**
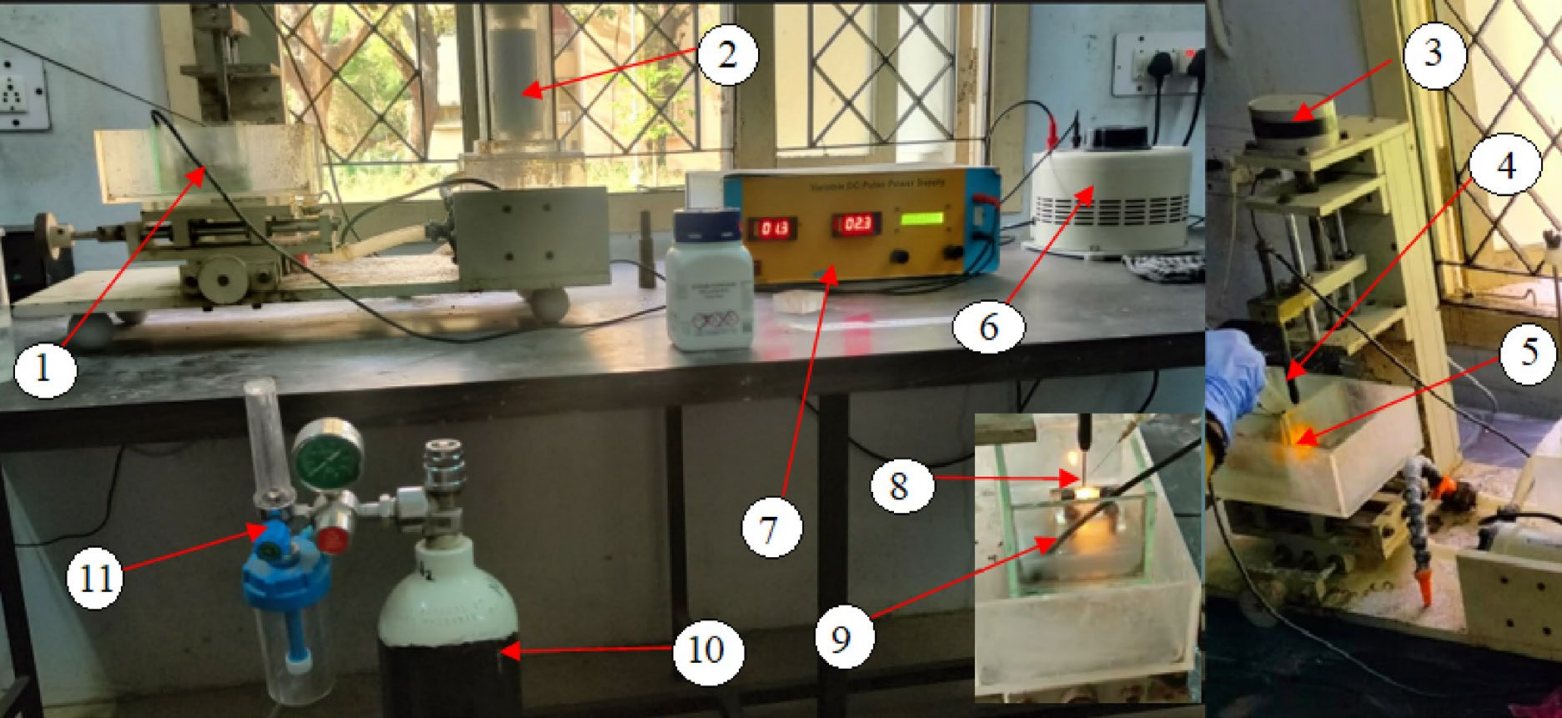
Indigenously developed ECDµM with di-electric gas setup. 1. Machining Chamber 2. Electrolyte filter 3. Stepper drive for cathode-electrode 4. Tool electrode holder 5. Spark generation 6. AC Voltage regulator 7. Gas flow regulator 8. Control unit for ECDµM 9. Auxiliary anode (Stainless Steel) 10. Clinical nitrogen cylinder 11. Pressure regulator and filter.

The ECDµM setup, as shown in Fig. 2, was ingeniously developed, capable of regulating voltage from 0 to 180 V through a control unit, which also provides precise control over frequency (3000–7000 Hz) and duty cycle ratio. The machining chamber, containing an aqueous NaOH electrolyte, is filled with controlled nitrogen gas, sourced from M/s. Akshaya Clinical-grade nitrogen gas supplier, Coimbatore. Nitrogen gas, readily available in the environment, not only stabilizes the gas film but also acts as a coolant during the machining process. Nitrogen gas was selected as the dielectric medium in this study due to its advantageous physical and chemical properties that enhance the ECDµM process. Compared to other gases such as argon, air or carbon di-oxide, nitrogen offers a stable and effective dielectric environment by maintaining a consistent gas film between the tool and workpiece, which is critical for spark generation and thermal management.

Nitrogen’s inert nature limits undesired chemical reactions during machining, reducing contamination risks and improving surface quality. Its higher thermal conductivity relative to argon and carbon di-oxide facilitates better heat dissipation in the machining zone, thus preventing excessive thermal damage while stabilizing MR and TW rates. Furthermore, nitrogen is more readily available and cost-effective than argon and safer compared to compressed air, which may contain moisture and reactive components that negatively affect machining stability^46^. Additionally, electrolysis generates a hydrogen gas film between the tool electrode and the workpiece. This gas film functions as a dielectric medium, impeding charge transfer. Once the applied voltage exceeds the critical threshold required to penetrate the gas layer and initiate a spark, thermal erosion begins on the workpiece, resulting in material removal.

The gas film formed during the process typically exhibits two regimes: discharge and hydrodynamic. Removal of material predominantly occurs in the discharge regime, particularly at depths below 300 µm^53^. However, as the machining depth exceeds 300 µm, the stability of the gas film diminishes in the hydrodynamic regime, leading to significantly reduced MR. Several factors, including the applied voltage, diameter of electrode, density of electrolyte, electrode material’s melting characteristics and spark intensity, are responsible for axial TW^54,55^. The careful selection of key influencing factors in ECDµM is crucial for achieving crack-free machined surfaces, minimizing the HAZ, reducing thermal fatigue, controlling the dielectric properties of the gas film, and maintaining uniform current density. RSM was applied to develop a mathematical model for identifying optimal machining parameters of a previous study on Hastelloy C276. The experimental results confirmed the robustness of the proposed multi-attribute optimization model, showing an average prediction error of only 5% compared to the experimental responses^56^. RSM enables efficient experimental planning and helps in identifying the interaction effects between variables. In this work, it was effectively used to study the influence of applied voltage, electrolyte concentration, and nitrogen gas flow rate on key responses such as MR and TW.

This methodology helps optimize the response functions, namely MR and TW, by identifying the optimal values of the independent variables^57^. The selected sample size was chosen to ensure reproducible and stable machining while allowing all process parameters to be effectively evaluated. The key factors—applied voltage, NaOH concentration, and nitrogen gas flow rate—exhibit strong interdependence, and the study aimed to optimize MR and TW simultaneously. To systematically investigate these interactions and identify the optimal parameter combinations, the experiments were designed using Central Composite Design (CCD) within the framework of RSM. CCD is a well-established statistical approach commonly employed in engineering research to model complex processes involving multiple interrelated input variables and to optimize performance outcomes. The chosen sample size ensures consistent and repeatable machining while accommodating all process parameters. For each parameter combination specified by the CCD, a single micro-cavity was produced on a 10 × 10 × 1 mm borosilicate glass specimen, and the MR and TW were determined from the difference in weight of the workpiece and the tool measured before and after machining.

The complete CCD matrix consisted of 20 distinct experimental conditions, which provides sufficient degrees of freedom for estimating the main effects, interaction terms, and quadratic terms in the RSM models while keeping the total machining time and specimen consumption at a practical level for ECDµM trials on brittle glass substrates^58^. The experiments were conducted using a domestically developed nitrogen gas-assisted ECDµM setup, operating at a constant current frequency of 5000 Hz and a duty cycle of 60%. Nitrogen gas was chosen for its high purity and clinical suitability, with pressure monitored and regulated via an attached pressure gauge. The nitrogen gas cylinder, with a 10-L capacity (1.5 m^3^), operated at a pressure of 150 psi.

To prevent the formation of NaOH monohydrates in the machining zone, the concentration of the aqueous NaOH electrolyte was kept below 40 wt% while maintaining controlled temperature conditions. The tool and workpiece were positioned at a minimal distance to initiate electrical sparks from the tool electrode to the generated gas film. Each machining experiment involved continuous machining of the borosilicate glass sample for 10 min to create a cavity. Nickel-coated mild steel was used as the cathode electrode (Ø 1 × 100 mm), while stainless steel (SS) served as the auxiliary electrode (12 × 6 × 3 mm) throughout the experiments. A notable feature of this ECDµM setup is, its non-short-circuit process, setting it apart from traditional ECM. The material loss from both the tool electrode and the borosilicate glass specimen was quantified using a high-precision electronic weighing scale with an accuracy of 0.001 g, measured before and after the machining process^30^. During the spark discharge process, the tool electrode emitted a significant number of electrons onto the borosilicate glass surface, generating localized heat and promoting material removal. The negative TW values recorded in Table 2 suggest an increase in the tool’s weight, likely due to the deposition of materials such as nitrides or byproducts from the machining environment. While this deposition would impact the final shape of the machined parts, it can also cause uneven sparking across the gap, leading to variations in the tool electrode’s electrical conductivity.

**Table 2:** Parameters and their Outcomes in ECDµM.

Std Order	Run Order	Pt Type	Blocks	Applied voltage (V)	Electrolyte Concentration (%wt)	Nitrogen gas flow rate (L/min)	MR (mg)	TW(mg)
17	1	0	1	100	20	4	4	4
10	2	− 1	1	134	20	4	5	− 1
1	3	1	1	80	10	3	1	1
7	4	1	1	80	30	5	0	0
2	5	1	1	120	10	3	0	1
18	6	0	1	100	20	4	0	− 1
15	7	0	1	100	20	4	1	1
6	8	1	1	120	10	5	2	− 1
5	9	1	1	80	10	5	2	1
13	10	− 1	1	100	20	2	2	2
20	11	0	1	100	20	4	1	1
9	12	− 1	1	66	20	4	1	3
11	13	− 1	1	100	3	4	0	− 1
3	14	1	1	80	30	3	1	1
19	15	0	1	100	20	4	2	3
14	16	− 1	1	100	20	6	1	0
4	17	1	1	120	30	3	4	3
8	18	1	1	120	30	5	4	2
12	19	− 1	1	100	37	4	5	5
16	20	0	1	100	20	4	1	0

Typically, in the hydrodynamic regime, the heat generated by electric discharge within the machining gap should aid in MR. Use of nitrogen gas enhances MR and reduces TW by preventing heat diffusion into the surrounding atmosphere. Additionally, the dielectric properties of nitrogen gas enhance the stability of the gas film in the machining zone. This strengthened gas film plays a crucial role in maintaining a consistent discharge environment, thereby improving machining efficiency and ensuring uniform material removal. Table 2 provides a summary of the parameter combinations and their corresponding results from the experiments. All reported values for MR and TW in this study represent the average of three independent experimental trials conducted under identical conditions. This averaging approach was adopted to ensure repeatability, minimize random errors, and enhance the robustness of the reported results.

All experimental data were obtained from the ECDµM setup using standard monitoring instruments for voltage, current, and nitrogen gas flow, together with a precision electronic balance to measure the weight of the tool and workpiece before and after machining. For each experimental run, the applied voltage, NaOH concentration, nitrogen flow rate, machining time, and the corresponding MR and TW values were recorded for analysis. The electrical parameters were monitored using calibrated meters integrated in the power supply and control unit, nitrogen flow was set and read using a commercially certified flow regulator, and weight measurements were taken with a 0.001 g resolution balance that was zeroed before each weighing and periodically checked with standard weights. These procedures were followed consistently to ensure that the collected data are reliable and based on valid, traceable measurements. The study does not involve human participants, animals, or proprietary clinical data, and only commercially available borosilicate glass and metallic tool materials were used; therefore, no specific ethical approval was required beyond adherence to institutional laboratory safety and environmental handling guidelines for electrolytes and gases. The effects of electrolyte concentration and nitrogen gas on MR and TW are clearly demonstrated in the experimental findings.

**Fig. 3:**
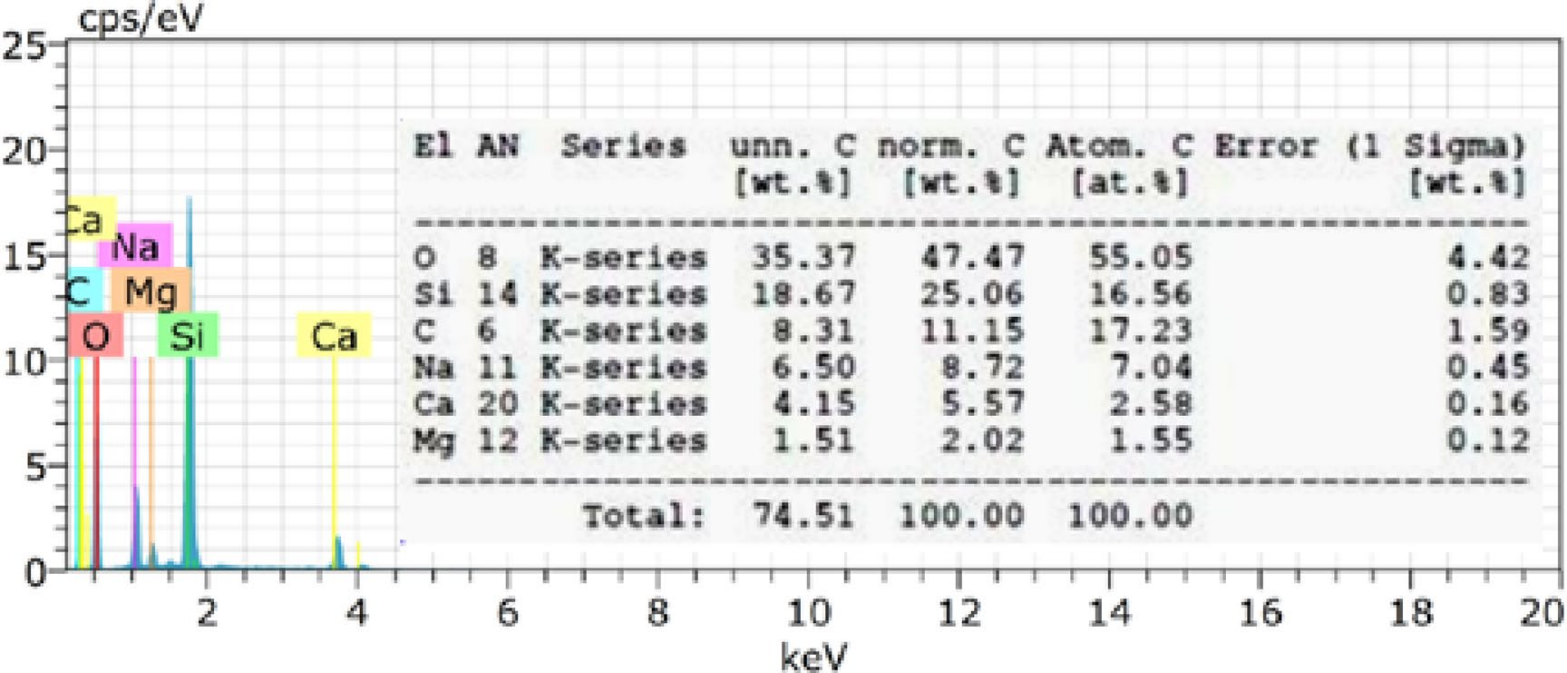
EDX- Borosilicate chemical composition.

Energy Dispersive X-ray Spectroscopy (EDX) is employed for rapid qualitative and quantitative analysis of the elemental composition of a material^59^. Hence, it is employed to analyze the chemical composition of the borosilicate glass specimen, revealing key elements like Si, Na, Ca, and Mg, as shown in Fig. 3. This EDX spectrum confirms the presence of major glass-forming and modifying oxides, particularly SiO₂, Na₂O, CaO, and MgO, which are characteristic of borosilicate glasses. Despite the absence of boron detection, the overall composition aligns well with the expected profile for borosilicate materials. These components contribute to the thermal shock resistance, chemical durability, and mechanical strength of the glass, making it suitable for applications in optics, labware, micro-machining and electronics.

## Results and discussion

The results have been presented by first examining the individual effects of applied voltage, NaOH concentration and nitrogen flow rate on MR and TW, followed by multi-response optimization using GRA and model validation with the RFA. This study explores the outcomes of creating micro-cavities on borosilicate glass specimens using clinical-grade pure nitrogen gas-assisted ECDµM with an aqueous NaOH electrolyte. The mechanism of MR is dominated by electrical spark discharges acting on the workpiece surface^60^. In contrast, material removal is driven by the combined effects of localized thermal energy and chemical etching^61^. MR mechanism can be described in terms of an arc discharge valve mechanism governing the discharge behavior^62^. Based on the findings of this study, MR is shown to occur due to localized material evaporation within the discharge and hydrodynamic regions of the gas film, in addition to chemical etching. The characteristics of the gas film play a critical role in sustaining a stable discharge in both regions, thus enhancing the efficiency of ECDµM.

### Effects of applied voltage on material removal (MR) and tool wear (TW)

**Fig. 4:**
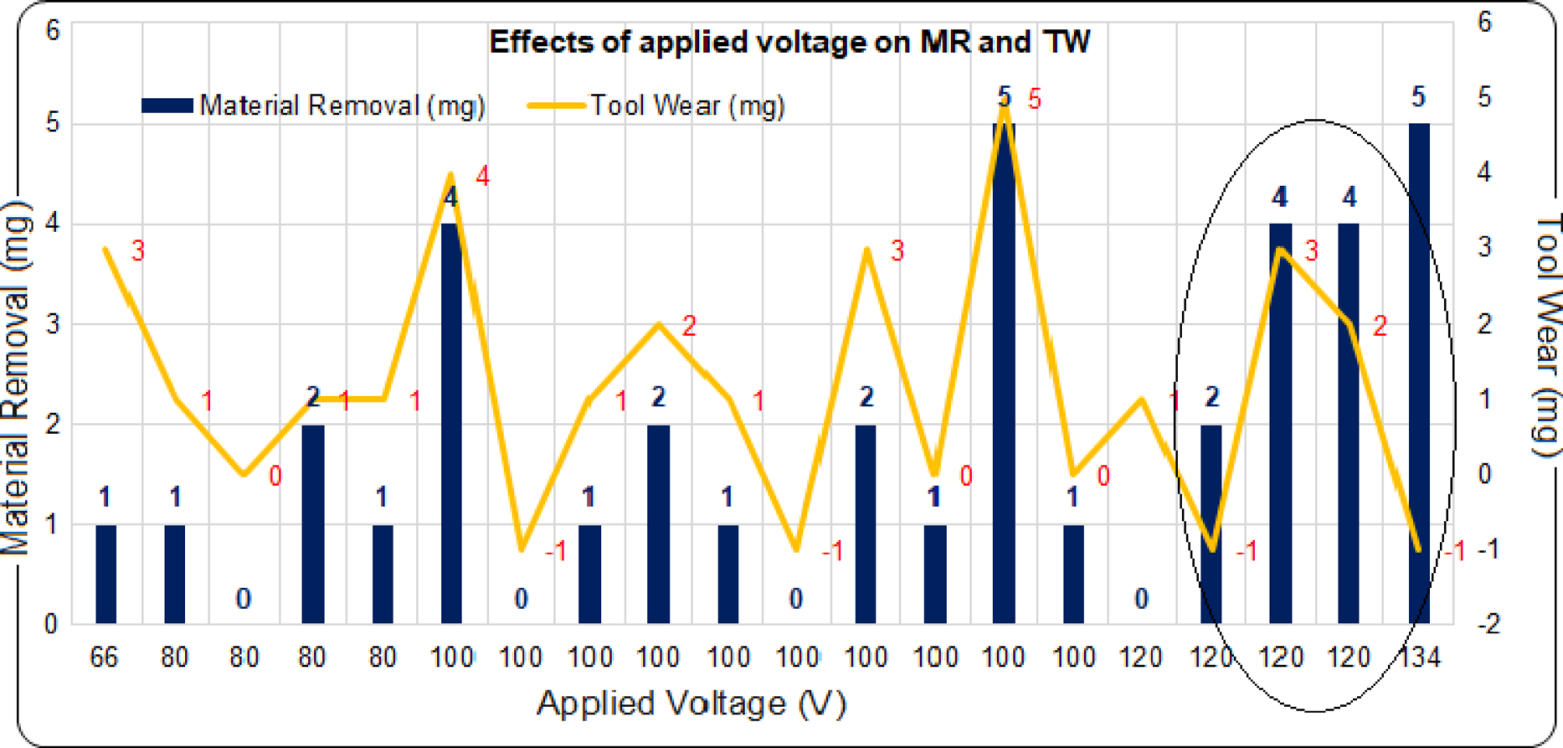
Effects of applied voltage on MR and TW.

Figure 4 demonstrates how applied voltage influences MR and TW during the machining of micro-cavities on borosilicate workpieces using nitrogen gas-assisted aqueous NaOH electrolyte in ECDµM. The insulating gas layer in the machining zone prevents the transfer of electric discharge energy to the workpiece, thus reducing thermal erosion and localized melting. As such, applying the right voltage to disrupt the gas film is essential, with the optimal value depending on factors like electrolyte concentration and the conditions conducive to bubble formation. Applied voltages between 66 and 134 V were applied according to the CCD of RSM, with MR and TW represented on both axes, highlighting the relationship between applied voltage and their combined effects. The phenomenon observed in ECDµM indicates that higher voltage generally results in greater MR, as supported by the data in Fig. 4. However, it was noted that a 120 V setting did not consistently produce optimal MR, suggesting that applied voltage alone may not be sufficient to achieve the desired outcomes. Based on the ANOVA, applied voltage exhibits a significant linear effect on MR, with an F-value of 7.60 and a p-value of 0.020, indicating that variations in voltage have a more pronounced impact on MR than TW.

Experimental results under varying electrolyte concentrations and nitrogen gas flow rates at 120 V showed different combinations of MR and TW. For example, a 30 wt% electrolyte concentration combined with a 5 L/min nitrogen gas flow rate led to a 100% increase in 4 mg of MR along with 2 mg of TW, though higher nitrogen gas flow rates caused tool weight gain due to the deposition of oxide layer. In contrast, better performance was observed at 100 V, where 5 mg of MR and 5 mg of TW were recorded under conditions of a 37 wt% electrolyte concentration and a 4 L/min nitrogen gas flow rate. Similarly, under the same voltage, a 20 wt% electrolyte concentration and a 4 L/min nitrogen gas flow rate resulted in 4 mg of MR and 4 mg of TW. These findings suggest that reducing the use of high electrolyte concentrations could help minimize soil pollution while still achieving optimal MR and TW. On the other hand, lower voltage settings, such as 80 V, failed to produce satisfactory results for achieving MR and TW. Therefore, selecting applied voltage above 100 V effectively disrupts the gas-insulation layer in the machining gap, enhancing MR. However, TW is influenced by electrolyte concentration and nitrogen gas flow rate, highlighting the interdependent nature of these parameters in optimizing both MR and TW.

### Effects of electrolyte concentration on material removal (MR) and tool wear (TW)

**Fig. 5:**
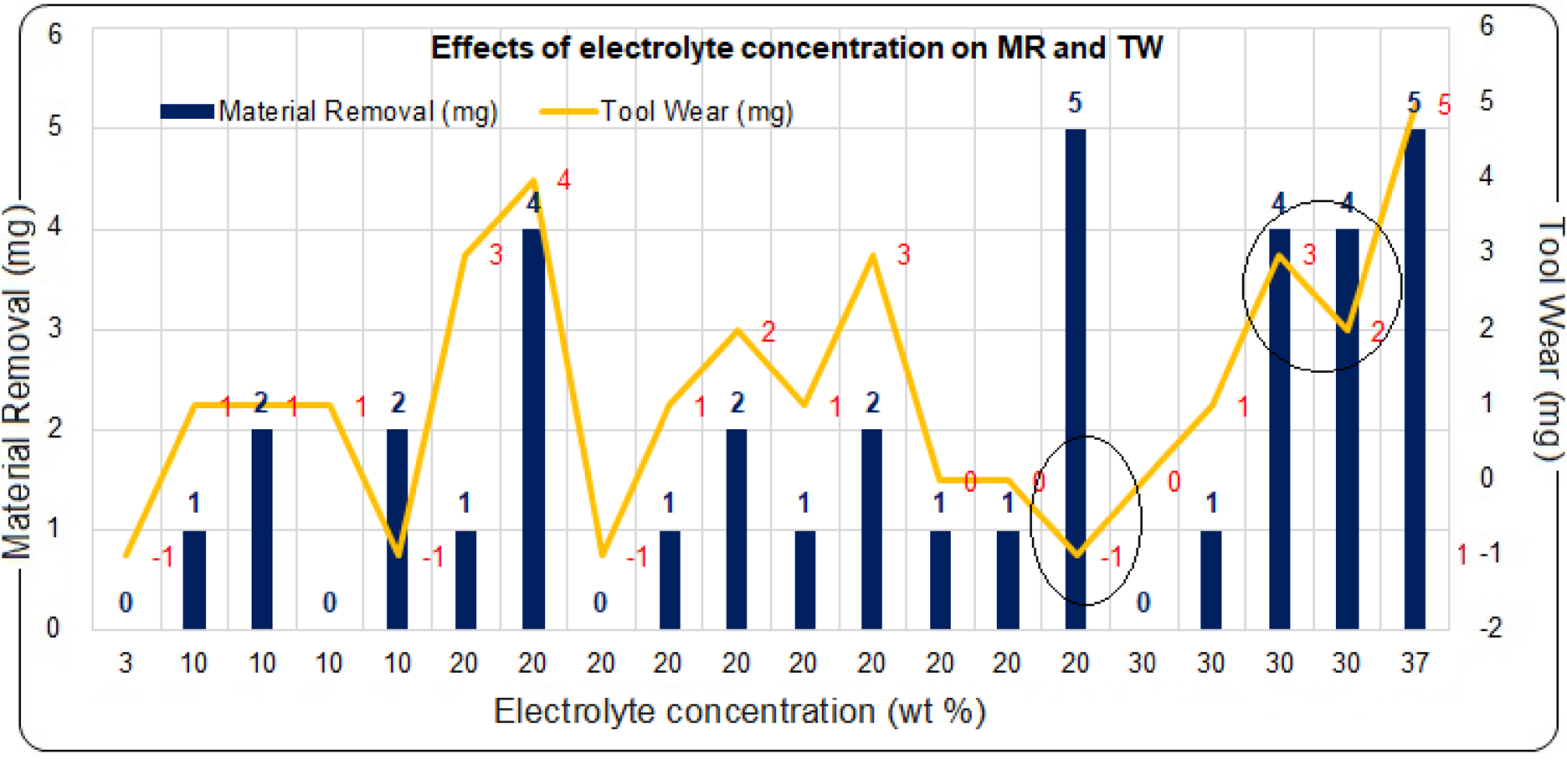
Effects of electrolyte concentration on MR and TW.

Figure 5 provides strong evidence that using a NaOH electrolyte concentration above 10 wt% enhances material removal, although other interdependent parameters also play a significant role in influencing tool wear. Three distinct scenarios were identified in the study.

Experimental findings indicate that a 20 wt% electrolyte concentration performs optimally, achieving concurrent MR and TW of 4 mg each under conditions of 100 V and 4 L/min. However, under the same concentration, MR of 5 mg and TW of − 1 mg are achieved under conditions of 134 V and 4 L/min gas-flow rate, representing a 25% increase in MR compared to 100 V. Essentially, NaOH is highly corrosive in nature, allowing for MR through corrosive action along with thermal erosion due to thermal fatigue load. However, the electrolyte concentration is limited to 40 wt% alone for better performance due to its solvent characteristics. Considering power consumption, 100 V yields superior MR compared to 134 V under the same electrolyte concentration and nitrogen gas flow rate. With respect to TW, the latter condition is more desirable, as TW debris can gradually alter the electrolyte concentration. Additionally, the accumulation of nitrides/byproducts on the electrode surface can adversely affect both thermal and electrical conductivity, leading to suboptimal discharge behavior. Consequently, the selection of the preferred condition is often guided by the industrial in-situ requirements. In the case of a higher electrolyte concentration of 37 wt%, higher MR and TW of 5 mg each are obtained under conditions of 4 L/min gas flow rate and 100 V. This represents a 25% increase in MR and a 150% increase in TW compared to results of 5 mg MR and 2 mg TW obtained under conditions of 134 V and 20 wt% electrolyte concentration. As per the ANOVA results, electrolyte concentration exerts a significant linear effect on MR, with an F-value of 7.25 and a p-value of 0.023, highlighting its critical role in MR. In the case of TW, electrolyte concentration exhibits the largest linear contribution among all factors, with an F-value of 4.80 and a p-value of 0.053, indicating a near-significant influence on thickness wear. It is concluded that above 100 V and 20–30 wt% electrolyte concentrations assist in achieving superior MR and TW simultaneously.

### Effects of nitrogen gas flow rate on material removal (MR) and tool wear (TW)

The nitrogen gas flow rate is a critical factor in the ECDµM process, significantly affecting both MR and TW. Experimental findings show that adjusting the nitrogen gas flow rate can cause substantial variations in MR and TW. Higher gas flow rates enhance MR by effectively dissipating the heat from the electric arc into the machining area, thus improving material removal. However, this can also lead to undesirable deposition on the cathode-electrode surface, which negatively impacts the tool’s characteristics, resulting in reduced MR and tool weight gain.

**Fig. 6:**
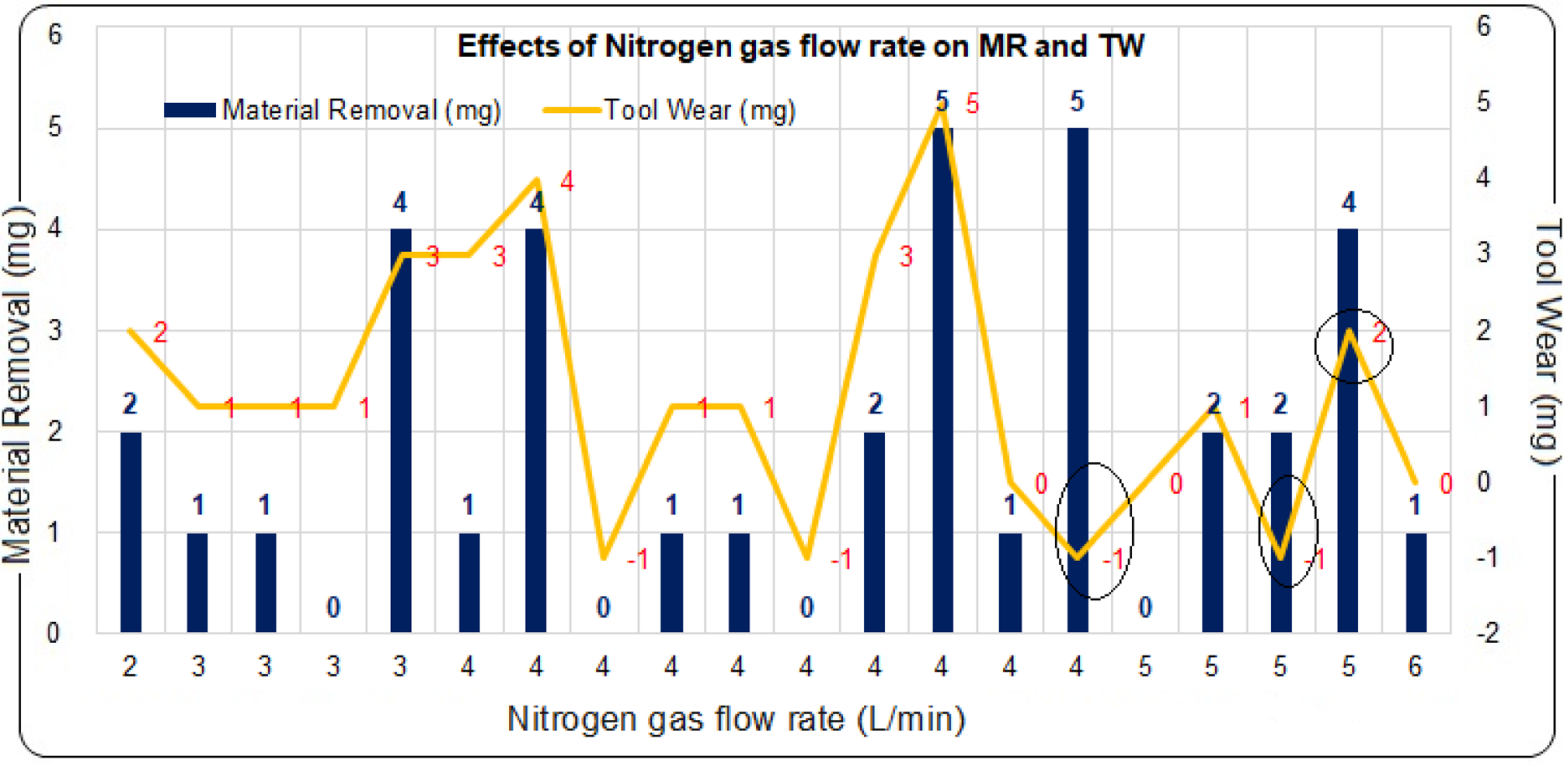
Effects of Nitrogen gas flow rate on MR and TW.

On the other hand, lower nitrogen gas flow rates reduce MR due to insufficient heat accumulation at the machining gap. Therefore, determining the optimal gas flow rate is essential to balance MR and TW. The influence of nitrogen gas flow rate on MR and TW is shown in Fig. 6. The best performance was observed with a gas flow rate of 4 L/min, where 5 mg of MR and − 1 mg of TW were achieved under 20 wt% electrolyte concentration and 134 V. Compared to 30 wt% NaOH, this represents a 33% reduction in electrolyte concentration, highlighting the role of nitrogen in enhancing process efficiency while minimizing chemical usage, reducing hazardous waste, and lowering environmental impact. At 120 V and 30 wt% NaOH, increasing the nitrogen gas flow rate from 3 L/min to 5 L/min maintained MR at 4 mg while reducing TW from 3 to 2 mg, confirming improved process stability and environmental benefits. This reduction in TW indicates lower energy consumption for tool regeneration and re-machining, which can be quantified as a 33% reduction in tool wear energy, reflecting improved energy efficiency in the process.

**Fig. 7:**
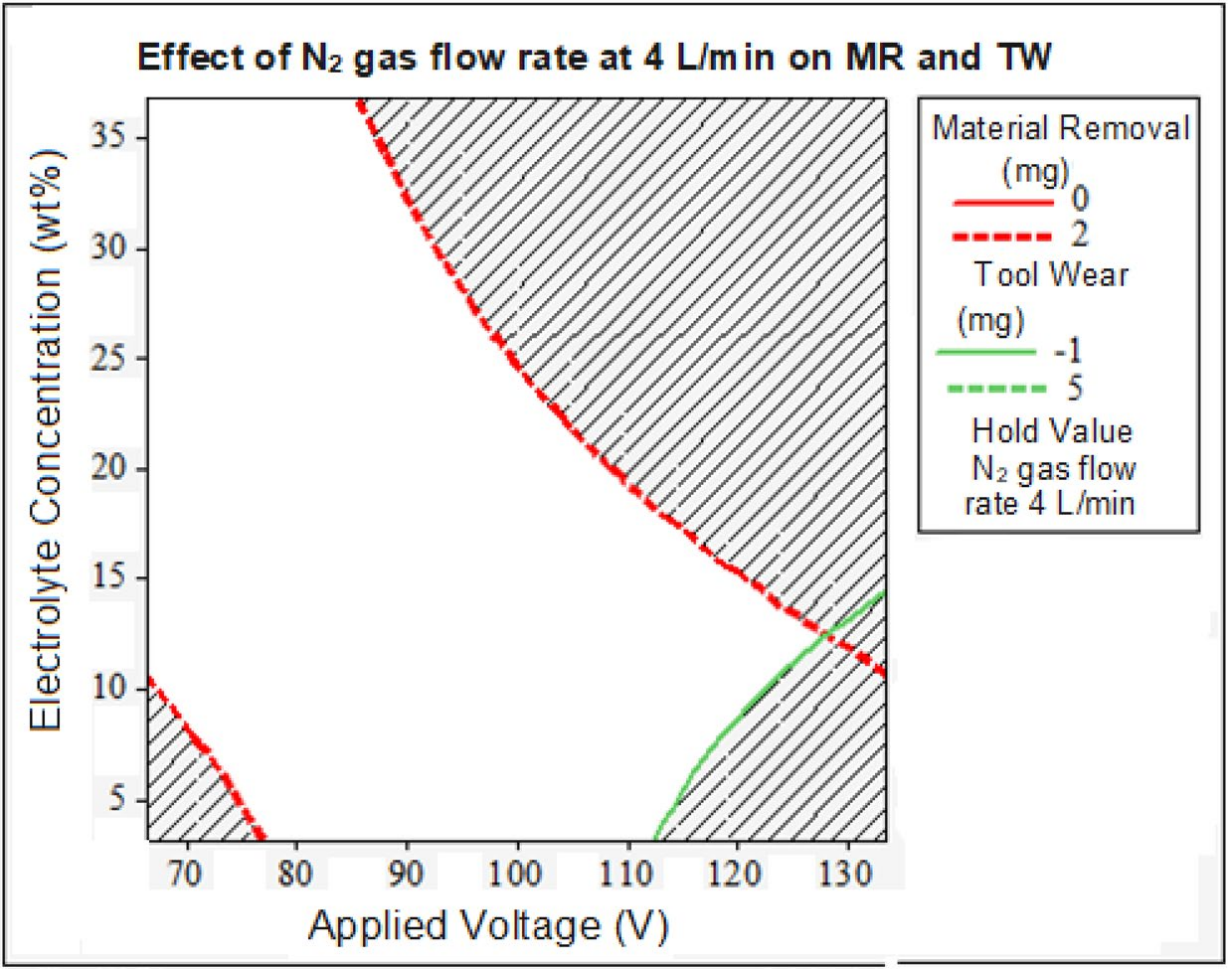
Optimal MR and TW Regions.

Similarly, with a nitrogen gas flow rate of 4 L/min, 5 mg of both MR and TW were achieved at applied voltage of 100 V with a 37 wt% electrolyte concentration. However, nitrogen gas flow rates of 5 L/min and 3 L/min were found to be less effective than the optimal flow rate of 4 L/min. These findings suggest that a nitrogen gas flow rate of 4 L/min provides the best performance under higher applied voltage conditions, particularly when the applied voltage exceeds 100 V and the electrolyte concentration is 20 wt% NaOH. The use of nitrogen gas promotes, as per ANOVA results, longer tool life, reduces the frequency of tool replacement and minimizes machining residues, directly leading to lower solid waste generation and a reduced ecological footprint. Figure 7 presents the parameter boundaries for identifying the optimal conditions for better MR and TW simultaneously. In the pursuit of an eco-friendly machining process, minimizing the use of higher electrolyte concentrations is a key goal. To identify the most effective parameters, RSM was employed. With the nitrogen gas flow rate fixed at the experimentally determined value of 4 L/min, the optimal region for achieving improved MR and TW is found at the intersection of the MR and TW lines, for applied voltage range of 100–125 V and the electrolyte concentration is above 10 wt%. These parameter ranges can be selected to simultaneously enhance MR and TW, with the final choice of parameters being tailored to meet specific industrial objectives.

### SEM images of micro-cavity and tool

**Fig. 8:**
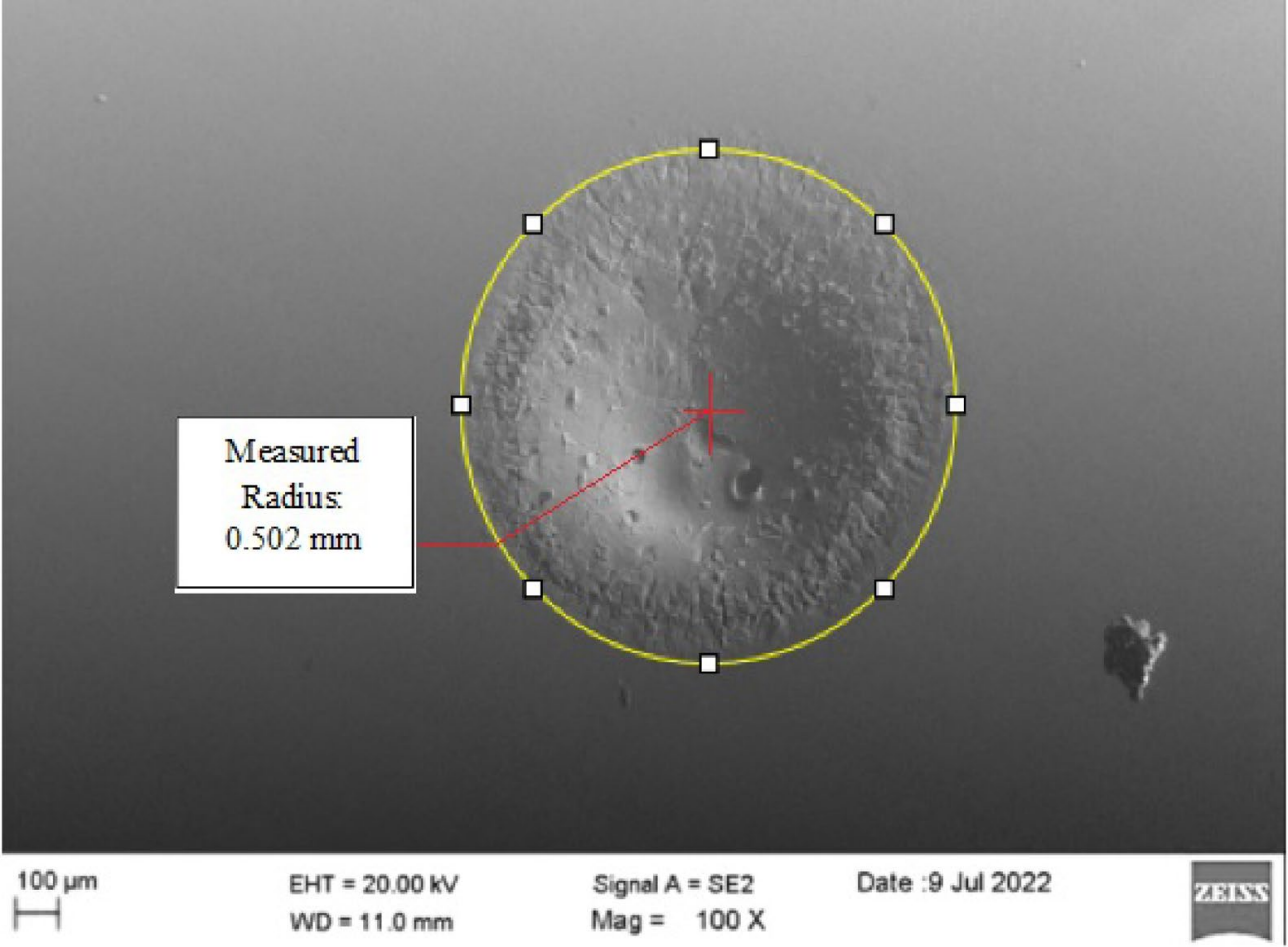
SEM image of micro-size cavity obtained at 120 V, 10 wt% and 5 L/min.

Figure 8 shows the micro-cavities formed on the borosilicate glass specimen, with a measured cavity radius of 0.502 mm, corresponding to a cavity diameter of 1.004 mm, which reflects a 0.4% deviation from the tool diameter. The Scanning Electron Microscope (SEM) image illustrates the material removal mechanism, where thermal fatigue induced by electric discharges caused localized chipping on the specimen surface. Nitrogen gas played a vital role in maintaining uniform current density across the machining gap, thereby ensuring stable material removal at the cavity center. At the specimen’s circumference, chemical and electrochemical reactions supported smoother material removal, resulting in improved surface finish. However, the brittleness of the glass made the surface prone to crack initiation from spark-induced chipping, underlining the importance of controlled machining parameters to achieve the desired cavity profile. Overall, the results demonstrate that the proposed method achieves finer surface quality, reduced thermal damage, and improved dimensional accuracy compared to conventional EDM-based methods.

**Fig. 9:**
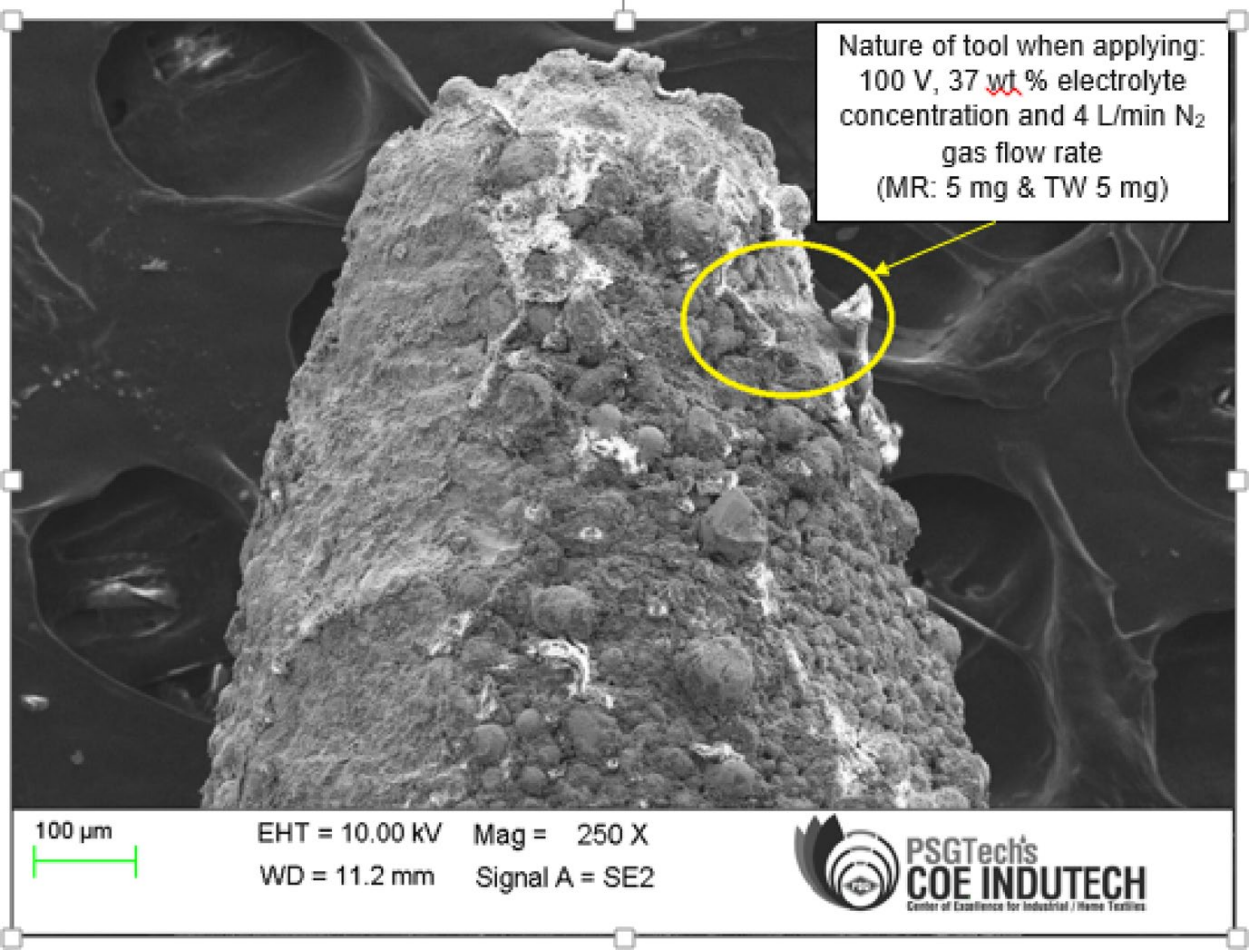
SEM image of tool at 100 V, 37 wt% and 4 L/min.

The SEM image of the tool obtained under the given conditions of 100 V, 37 wt% electrolyte concentration, 4 L/min N₂ gas flow rate is presented in Fig. 9.

The tool surface shows a rough and irregular texture with significant deposition and cluster-like structures as highlighted in the yellow circled area. The spherical and granular formations due to chemical reactions and agglomeration during machining. The uneven surface and accumulation suggest that localized TW (loading –negative wear) and thermal-chemical interactions occurred. Notably, at lower electrolyte concentrations compared to 37 wt%, the use of nitrogen gas as a dielectric medium is expected to further enhance eco-friendliness and reduce secondary waste formation, compared to kerosene or DI water systems.

### Sustainability considerations of nitrogen gas assisted ECDµM

A recent review outlines various sustainable machining technologies, with particular focus on nitrogen-assisted machining. It emphasizes nitrogen’s advantages as a dielectric medium—enhanced tool life and reduced environmental impact—compared to conventional fluids such as kerosene and DI water^63^. Although liquid nitrogen in cryogenic machining improves tool life and energy efficiency, its high consumption, storage demands, and liquefaction energy costs limit sustainability. In contrast, gaseous nitrogen applications offer simpler handling, lower resource use, and greater environmental benefits^64^. Nitrogen gas assisted ECDµM utilizes nitrogen as a dielectric medium, offering significant environmental and operational advantages over traditional dielectrics like kerosene or liquid nitrogen. Being abundant, inert, and non-toxic, nitrogen gas reduces ecological impact compared to hydrocarbon-based fluids. Its gaseous state ensures stable discharge conditions while limiting contamination of the dielectric, which helps maintain tool life and surface quality. Unlike liquid dielectrics, nitrogen does not generate hazardous effluents, supporting cleaner and more sustainable manufacturing processes.

The low viscosity and high insulating properties of nitrogen gas lower specific cutting energy, increasing machining efficiency. Its high thermal conductivity stabilizes the gas film, reduces excessive heat, and enhances precision while lowering energy demands for subsequent surface corrections. Nitrogen gas also facilitates rapid debris removal, reducing waste buildup and prolonging dielectric usability. By integrating nitrogen gas assisted ECDµM with advanced process control, such as machine learning techniques, resource-efficient micro-machining of brittle materials like borosilicate glass becomes possible. Overall, nitrogen gas provides a clean, energy-efficient, and sustainable approach, aligning with the principles of green manufacturing and circular economy practices.

## Response surface methodology (RSM)

RSM is an essential tool for optimizing machining processes by providing a structured approach for experimentation and process behavior analysis. RSM enables the systematic investigation of the relationship between input parameters (factors) and output responses in machining processes. In this study, RSM was employed to identify the relationship between the factors and the selected objectives^57,65^. After completing the experimental phase, the next step involves using the input vector (X) and corresponding outputs (Y) to fit a suitable model, which predicts the process responses based on the experimental design. Typically, a mathematical polynomial function is selected as the response surface model. Equation 1 illustrates the general second-order polynomial response surface model, which evaluates the effects of various factors on different response criteria.

1 $$\begin{aligned} {\mathrm{Y}} & = \upbeta_{0} + \upbeta_{{1}} *{\mathrm{X}}_{{1}} + \upbeta_{{2}} *{\mathrm{X}}_{{2}} + \cdots + \upbeta_{{{11}}} *{\mathrm{X}}_{{1}}^{{2}} \\ & \quad + \upbeta_{{{12}}} *{\mathrm{X}}_{{1}} *{\mathrm{X}}_{{2}} \, + \,\upbeta_{{{22}}} *{\mathrm{X}}_{{2}}^{{2}} \, + \cdots + \,\varepsilon \\ \end{aligned}$$

Here,

Y is the response variable (output), X_1_, X_2_, …, X_n_ are the input variables (factors), β_0_, β_1_, β_2_, …, β_11_, β_12_, β_22_, …, are the coefficients of the model representing the linear, quadratic, and interaction effects, ε is the error term.

The developed mathematical models, using Minitab V17, were presented in Eqs. 2 and 3. These models allow to analyze the effects of individual factors, their interactions, and quadratic effects on the response variables. The consistency of the model have been checked by the conduction of confirmatory experiments. The results revealed that the deviation of the confirmatory results from the model are found to be less than 4%.

2 $$\begin{aligned} MR \left( {mg} \right) & = 15.71 - 0.3*X_{1} - 0.297*X_{2} \\ & \quad + 0.263*X_{3} + 0.001*X_{1}^{2} \\ & \quad + 0.002*X_{2}^{2} - 0.064*X_{3}^{2} + 0.005*X_{1} *X_{2} \\ & \quad + 0.013*X_{1} * X_{3} - 0.05*X_{2} *X_{3} \\ \end{aligned}$$

3 $$\begin{aligned} TW \left( {mg} \right) & = - 0.568 + 0.0526*X_{1} - 0.335*X_{2} \\ & \quad + 1.722*X_{3} - 0.0005*X_{1}^{2} + 0.002*X_{2}^{2} \\ & \quad - 0.121*X_{3}^{2} + 0.0038*X_{1} *X_{2} - 0.013* X_{1} *X_{3} \\ \end{aligned}$$

where *X*_*1*_ = Applied Voltage (V); *X*_*2*_ = Electrolyte Concentration (wt%); *X*_*3*_ = N_2_ gas flow rate (L/min)

**Fig. 10:**
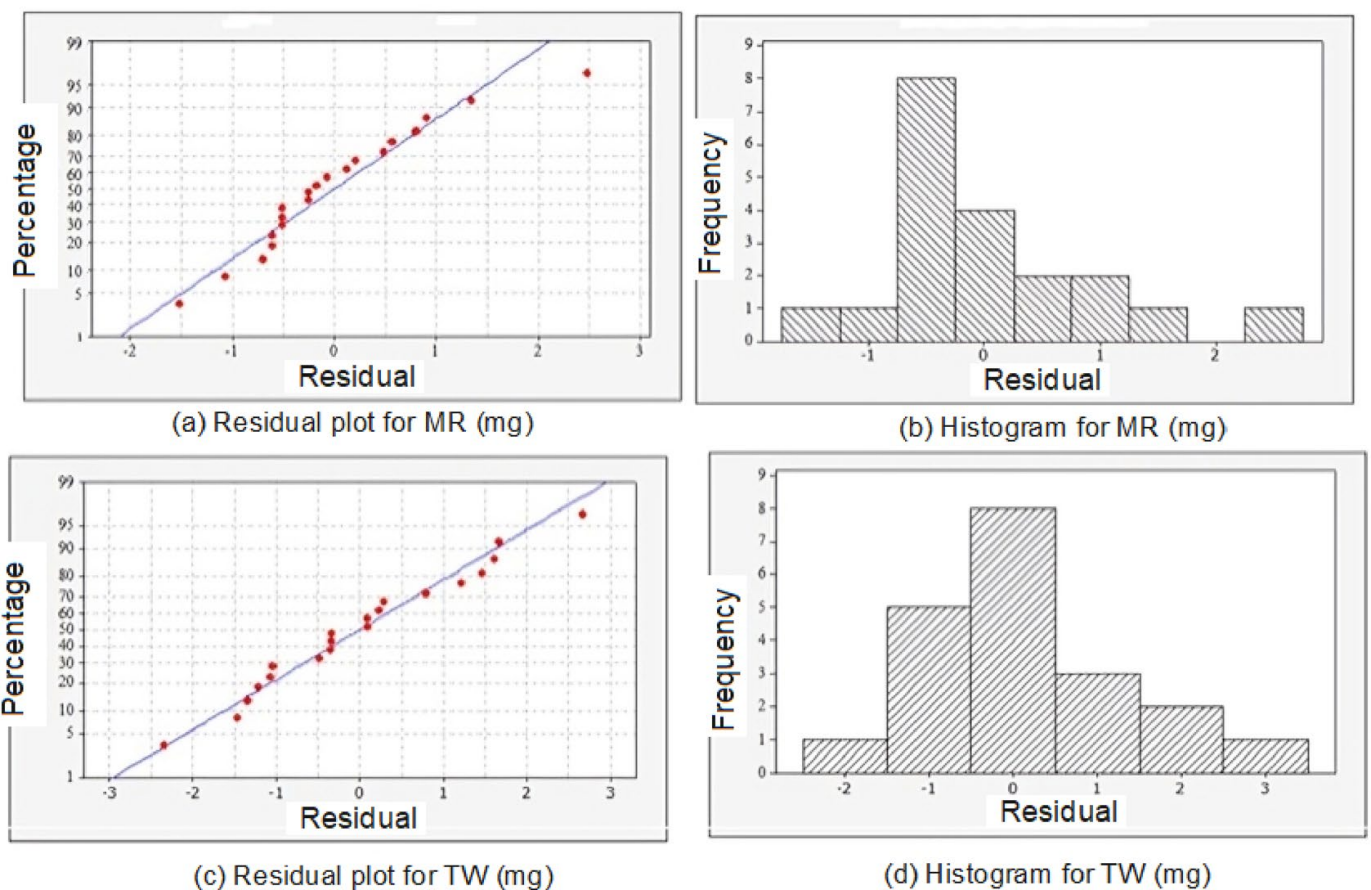
Residual and Histogram plots for MR and TW.

In RSM, a normal probability plot serves as a graphical tool to evaluate the normality of residuals in the fitted regression model. This assessment is vital for ensuring that the assumptions of normality are satisfied, which is necessary for the validity of the statistical inferences derived from the model. Figure 10 presents the probability and histogram plots for MR and TW. The residual plots in Fig. 9a and c show the experimental performance trends compared to the predicted values. Both plots display random scatter within a small boundary, indicating a good fit between the observed and predicted results. However, the histogram plots in Fig. 10b and d highlight the differences between the predicted and actual values. Figure 10 b shows the histogram for MR to be right-skewed, indicating a positive skewness. This suggests that while most of the MR values are clustered towards the lower end, there are a few higher MR values. The peak of the distribution is on the left, and the mean is greater than the median, which in turn is greater than the mode (Mean > Median > Mode), indicating a positive skew. Extreme observations can contribute to this skewness. If further refinement is needed, increasing the dataset size can help reduce skewness and stabilize the distribution. Similarly, the TW residual plots and histogram (Fig. 10c and d) exhibit a similar trend. It is also observed that the degree of difference between the mean, median, and mode varies within the right-skewed distribution, as seen in the pattern of the histogram in Fig. 10d when compared to Fig. 10b.

**Fig. 11:**
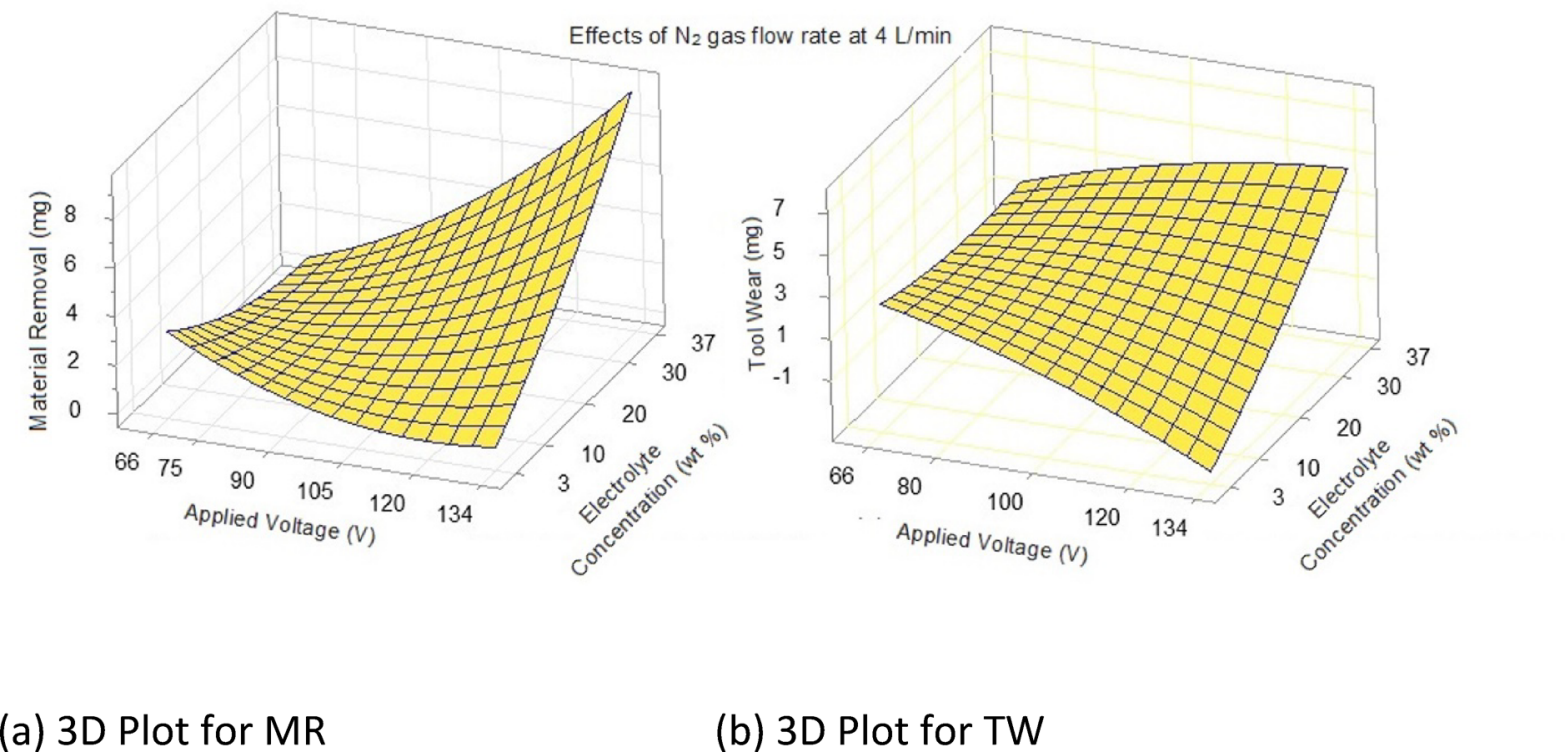
Predicted 3D contour plots for MR and TW.

This observation suggests that the distribution of TW residuals may exhibit more pronounced skewness than the MR residuals. Analyzing the residual plots and histograms offers valuable insights into the performance of the experimental model, revealing any discrepancies between predicted and actual values. This analysis also helps in understanding the nature of the residual distributions for each response variable. The 3D contour plots for MR and TW, predicted using Minitab V17 based on the established Eqs. (2 and 3), are presented in Fig. 11.

To achieve optimized MR and TW, equal importance is assigned to both, with each receiving a 50% weightage^49^. Figure 11a and b show the predicted 3D contour plots, which are consistent with the established relationship between applied voltage, electrolyte concentration, MR and TW, at a nitrogen gas flow rate of 4 L/min.

## Grey relational analysis (GRA)

GRA is a decision-making method used to evaluate and rank alternatives based on multiple criteria. For this work employed GRA, which is well-suited for multi-objective optimization where responses have conflicting goals. GRA provides a normalized and weighted evaluation of multiple performance indices, allowing for an effective compromise solution that balances MR and TW. The combined use of RSM and GRA enhances the reliability and accuracy of the optimization process. Originating from Grey system theory, it is particularly useful for analyzing systems with incomplete or uncertain information. GRA combines both subjective and objective data to make decisions, making it ideal for scenarios where data is sparse or uncertain. In GRA, each alternative is assessed across multiple criteria, and performance scores are assigned based on subjective assessments or available data. These scores are processed through mathematical operations to determine the final ranking of alternatives^35,36^.

The GRA method helps reduce uncertainty and subjectivity in decision-making by providing a structured and transparent way to evaluate alternatives. In the context of ECDµM, GRA is applied to identify the feasible values of the influencing factors. The results in Table 3 show the combination of 134 V, 20 wt% and 4 L/min flow rate achieved the highest ranking among the 20 different combinations evaluated. Rankings also indicated that applied voltages ranging from 100–120 V, electrolyte concentrations between 10 wt% and 20 wt%, and nitrogen gas flow rates of 4–5 L/min obtained the second and third highest ranks. These findings highlight the importance of customizing parameter sets to meet specific needs and demonstrate the interconnectedness of factors influencing the micromachining process. The integration of nitrogen gas played a key role in enhancing MR while minimizing TW by preserving arc-discharge characteristics within the machining zone. The identified parameter sets, supported by experimental data, underscore the role of nitrogen gas in improving process efficiency.

**Table 3:** GRA and its ranking.

Exp.No	Applied Voltage V	Elect. con wt%	N_2_ gas flow rate (L/ min)	Output Responses		Normalizing Sequence		Grey Relational Coefficient		Grey Rational Grade (GRG)	Weighted GRG (50:50)	GRG Rank
				MR (mg)	TW (mg)	MR (mg) Max	TW (mg) Min	MR (mg)	TW (mg)			
1	66	20	4	1	3	0.20	0.333	0.384	0.428	0.407	0.402	20
2	80	10	5	2	1	0.40	0.667	0.454	0.599	0.527	0.513	12
3	80	30	3	1	1	0.20	0.667	0.384	0.599	0.492	0.471	13
4	80	30	5	0	0	0.00	0.833	0.333	0.749	0.541	0.500	11
5	80	10	3	1	1	0.20	0.667	0.384	0.599	0.492	0.471	13
6	100	20	4	2	3	0.40	0.333	0.454	0.428	0.441	0.444	19
7	100	3	4	0	− 1	0.00	1.00	0.333	0.998	0.666	0.599	4
8	100	20	4	4	4	0.80	0.167	0.714	0.374	0.545	0.579	10
9	100	37	4	5	5	1.00	0.00	1	0.333	0.667	0.733	3
10	100	20	4	1	0	0.20	0.833	0.384	0.749	0.567	0.530	8
11	100	20	4	1	1	0.20	0.667	0.384	0.599	0.492	0.471	13
12	100	20	4	0	− 1	0.00	1.00	0.333	0.998	0.666	0.599	4
13	100	20	2	2	2	0.40	0.50	0.454	0.499	0.477	0.473	17
14	100	20	6	1	0	0.20	0.833	0.384	0.749	0.567	0.530	8
15	100	20	4	1	1	0.20	0.667	0.384	0.599	0.492	0.471	13
16	120	30	5	4	2	0.80	0.500	0.714	0.499	0.607	0.628	6
17	120	10	3	0	1	0.00	0.667	0.333	0.599	0.466	0.440	18
18	120	30	3	4	3	0.80	0.333	0.714	0.428	0.571	0.600	7
19	120	10	5	2	− 1	0.40	1.00	0.454	0.998	0.726	0.672	2
20	134	20	4	5	− 1	1.00	1.00	1	0.998	0.999	0.999	1

## Random forest algorithm (RFA)

The RFA was applied as an ensemble-based machine learning technique to predict machining responses and to validate the optimal conditions obtained through GRA. Unlike metaheuristic algorithms, RFA is not an optimization tool; rather, it is a predictive model trained on experimental data. The algorithm generates multiple decision trees during training and combines their outputs to enhance accuracy and reliability. In a random forest, each tree is constructed using a randomly sampled subset of the available data, and node splitting is carried out using randomly selected features. This approach reduces overfitting and improves generalization performance. As the number of trees increases, the overall error rate converges and stabilizes, depending on the predictive strength of individual trees and the correlation between them.

**Fig. 12:**
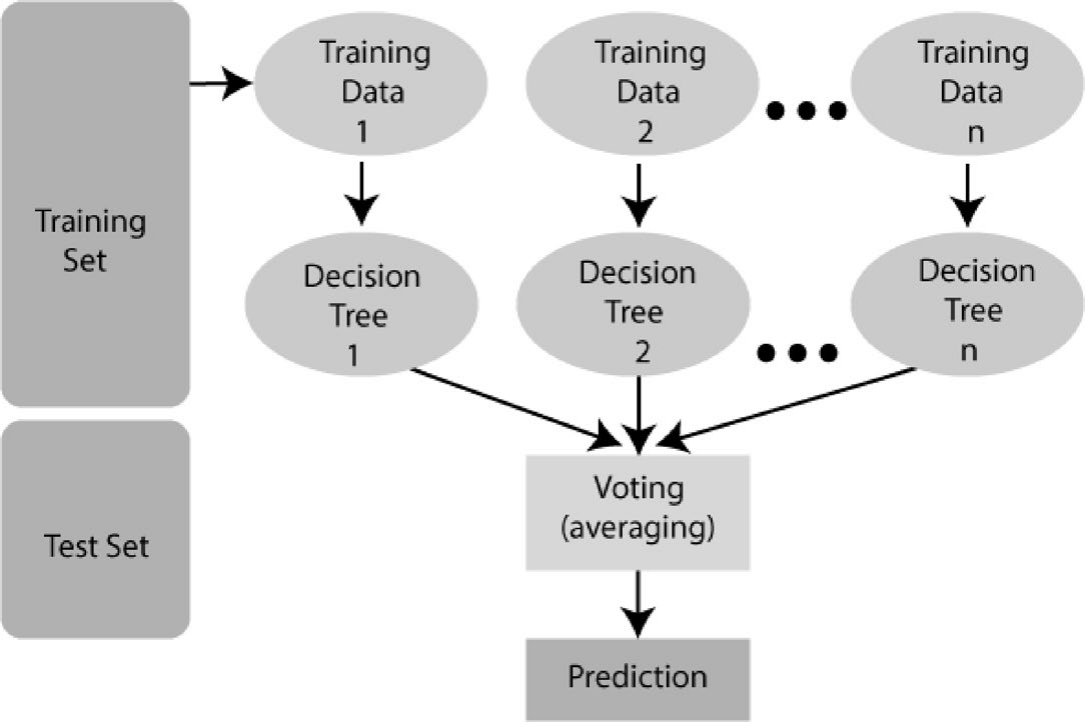
Working mechanism of RFA.

Random feature selection during the splitting process also enables the model to achieve error rates comparable to Adaboost while offering superior robustness against noise. In addition, the algorithm provides internal error estimates and measures of variable importance, making it possible to assess the influence of different process parameters and to evaluate the effect of increasing the RFA in modeling complex machining processes. The workflow of RFA applied in this number of features during training. This predictive capability reinforces the reliability study is presented in Fig. 12.

In this study, 500 trees were used, as increasing the number beyond this value produced negligible improvement in prediction accuracy. The maximum depth of each tree was set to “none” (allowing full growth), while the minimum number of samples required to split an internal node was set to 2. The Gini impurity criterion was adopted for node splitting. RFA reduces overfitting by aggregating multiple decision trees, making it more reliable than individual models such as ANN or Support Vector Machines (SVM), especially with limited experimental data.

**Fig. 13:**
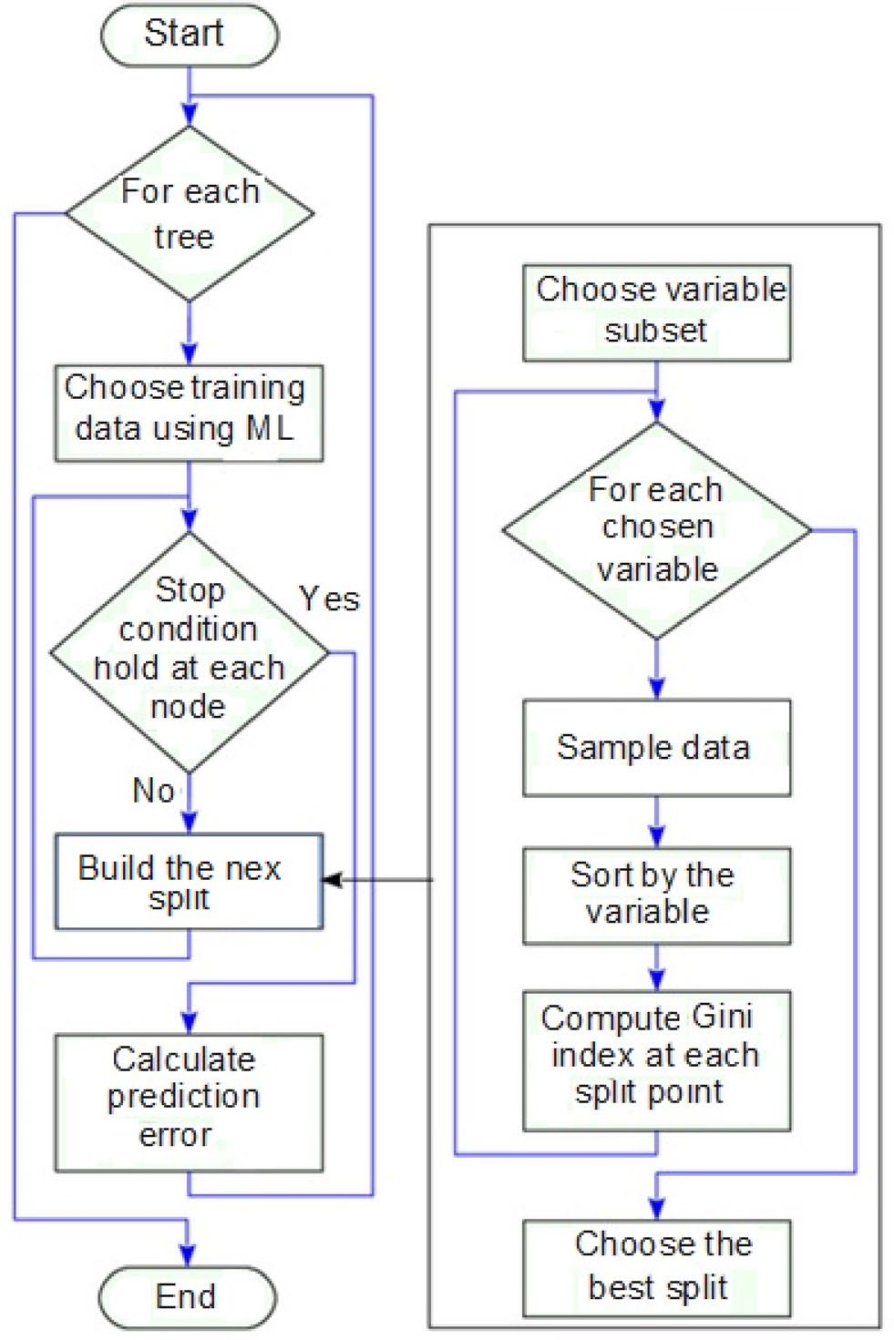
Functional flowchart for RFA.

Furthermore, RFA effectively handles nonlinear and intricate interactions among machining parameters, which are common in ECDµM processes. It also provides interpretable outputs through feature importance rankings, aiding in the identification of influential parameters. In machine learning, a hyperparameter is a setting chosen before training the model that controls how the algorithm learns from data. Unlike model parameters like the split points inside each tree, which are learned during training, hyperparameters must be set by the user or tuned through methods like grid search or cross-validation. Compared to other models, RFA requires less hyperparameter tuning and converges rapidly, making it well-suited for experimental prediction and validation scenarios^66^. In this study, RFA is used to identify the influencing factors for improving MR and TW simultaneously in the ECDµM process for borosilicate glass specimens. The RFA combines multiple decision trees to predict the class of the dataset. While individual trees may make incorrect predictions, the collective output of all trees tends to be accurate^42,66^. The RFA functioning flowchart is presented in Fig. 13. To ensure optimal recital of a random forest classification mechanism, two key assumptions should be considered namely: The feature variables in the dataset should contain actual values rather than guessed ones to enable precise predictions and predictions from each tree would have minimal correlation with each other. The random forest prediction can be found using Eq. 4.

4 $${\text{Random Forest prediction }} = \frac{1}{k}\mathop \sum \limits_{k = 1}^{k} h_{k} \left( x \right)$$

Here, *K* represents the quantity of autonomous regression trees generated for the bootstrap samples with input vector x and hk(x) denotes the average of predictions produced by K regression trees.

Equation 5 is utilized to compute the mean squared error for out-of-bag data dataset.

5 $${\mathrm{MSE}}_{{{\mathrm{OOB}}}} = \frac{1}{n}\mathop \sum \limits_{i = i}^{n} \left( {y_{i} - \overline{y}_{iOOB} } \right)^{2}$$

where *y*_i_ and $\overline{y}_{iOOB}$ are the *i* th prediction and the mean of *i* th prediction from all the trees. Equation 6 is employed to calculate the coefficient of determination for the out-of-bag data dataset^42^.

6 $$R_{OOB}^{2} = 1 - MSE_{OOB} /Var_{y}$$

**Fig. 14:**
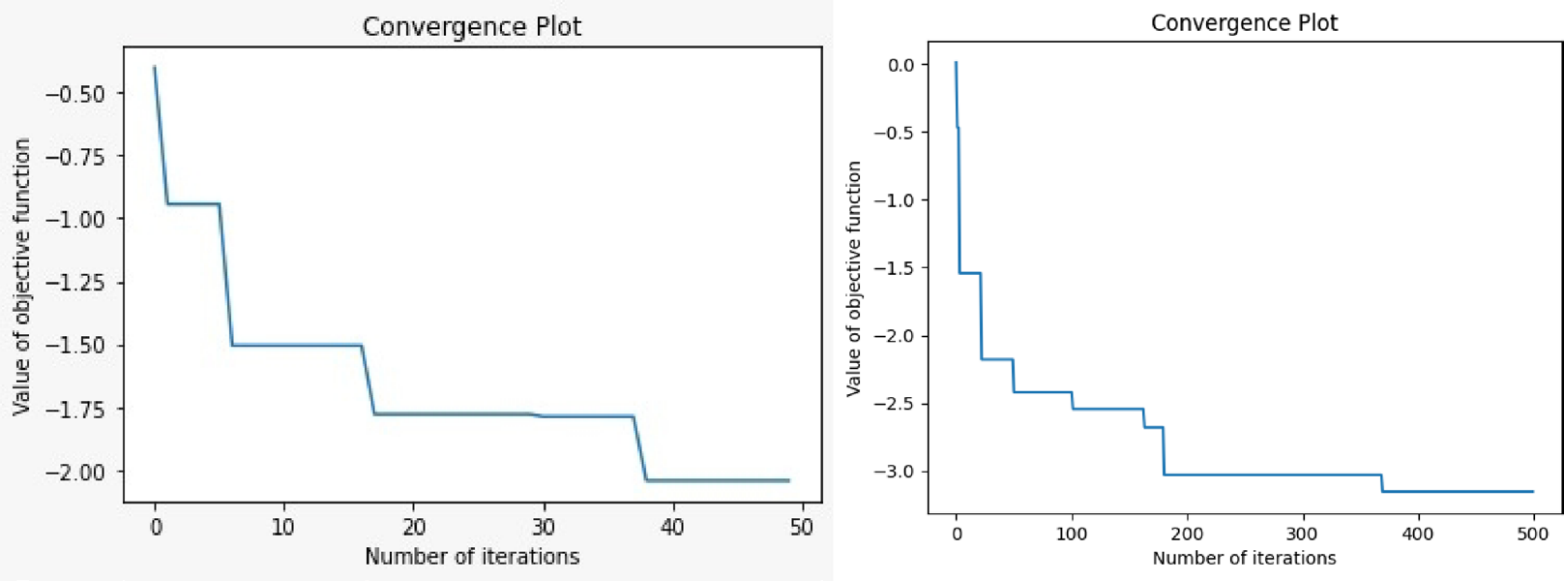
Convergence plot for RFA with different iterations.

A convergence plot of a nature-inspired RFA illustrates how the algorithm progresses over iterations towards an optimal solution. In general, the objective function’s value is displayed on the y-axis, while the x-axis indicates the number of iterations. As the algorithm iterates, the objective function value ideally decreases in the case of minimization problems or increases for maximization problems, indicating improvement in the solution’s quality. The convergence plot helps analysts understand the algorithm’s behavior, including its speed of convergence, stability, and whether it reaches a satisfactory solution. The obtained convergent plot for RFA is presented in Fig. 14.

The convergence plot illustrates that as the iterations increase, the optimization algorithm approaches convergence, indicating stability and consistency in the optimization process. This convergence behavior suggests that higher numbers of iterations are beneficial for accurately identifying the optimal values of the influencing parameters. As the algorithm iterates through successive steps, it refines its search and gradually hones in on the most promising regions of the parameter space, eventually resulting in the identification of the best options.

**Fig. 15:**
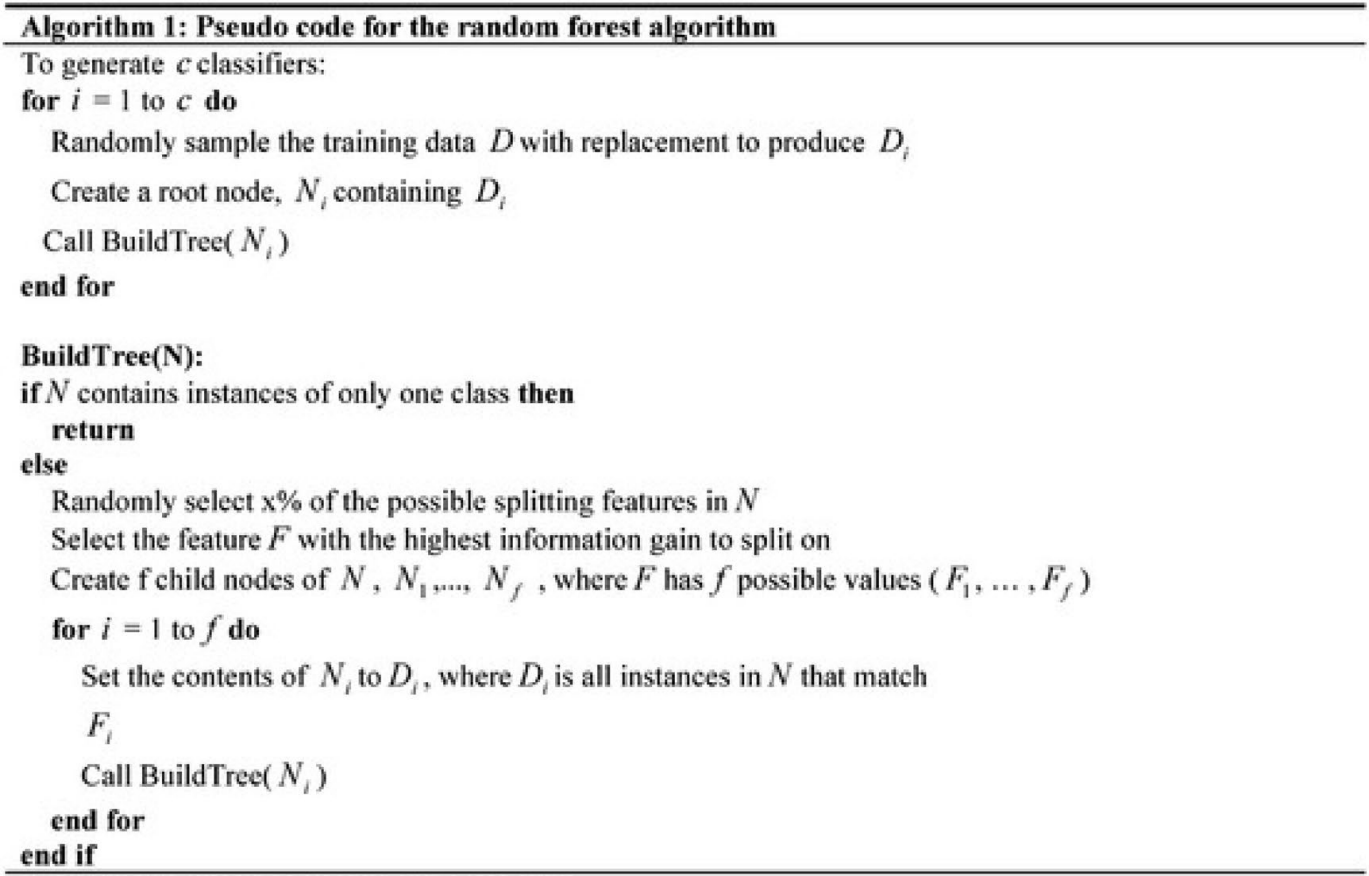
Pseudo code for RFA.

In essence, the convergence plot gives appreciated understandings into the performance of the RFA, demonstrating how the objective function value changes over time and indicating when the algorithm has reached a satisfactory solution. The nature of training influences the convergence, which is concretely evidenced in Fig. 14. The number of iterations influences the convergence characteristics of the RFA and helps obtain optimal values, particularly when uncertainty arises in the selection of influential factors. It enables manufacturers to understand the relationships between process parameters and outcomes without extensive trial-and-error experiments. Additionally, it can handle large datasets and complex, non-linear relationships, making it applicable across various materials and machining setups. The pseudo code outlines the general structure of an optimization algorithm, including the initialization of the population, the main loop where evolution occurs, and the termination condition. The pseudo code of the RFA is shown in Fig. 15.

## Performance evaluation

The performance of the RFA was evaluated against several widely used metaheuristic algorithms, including the Genetic Algorithm (GA)^67^, Particle Swarm Optimization (PSO), Teaching–Learning Based Optimization (TLBO) and Sparrow Search Algorithm (SSA)^68–76^. These algorithms were chosen due to their effectiveness in optimization tasks and their established applications in machining process optimization. The parameter configurations for these algorithms were adopted from prior research studies and are detailed in Table 4.

**Table 4:** Parameters of the selected Metaheuristic Algorithms.

Algorithm	Control Parameters
GA	Number of Iterations: 50, Seed = 1, population size: 50, ${P}_{mut}=0.9$
PSO	Number of Iterations: 50, Size of Swarm: 50, $\alpha =0.2$*, β* = *γ* = *1*
TLBO	1. Population Size: 25 (npop_o) & Number of Generations: 50 2. Teaching Factor: Randomly chosen between 1 and 2 during the update process 3. Randomization: Randomization is employed in selecting individuals and generating random numbers used in the TLBO algorithm
SSA	1. Number of Sparrows: It is set to ‘num_sparrows = 30’ 2. Adjustment elements (a and b): They are set to ‘a = 2’ and ‘b = 1’ 3. Bounds: The bounds are defined as `bounds = np.array ((80, 120), (10, 30), (3, 5))
RFA	No. of Iterations: 50, Maximum Depth (max_depth): None (allow trees to grow fully), Maximum Features (max_features): 1.0, Least -Samples Split (min_samples_split): 2 Least- Samples Leaf (min_samples_leaf): 1 Random State: 1 (for reproducibility)

**Fig. 16:**
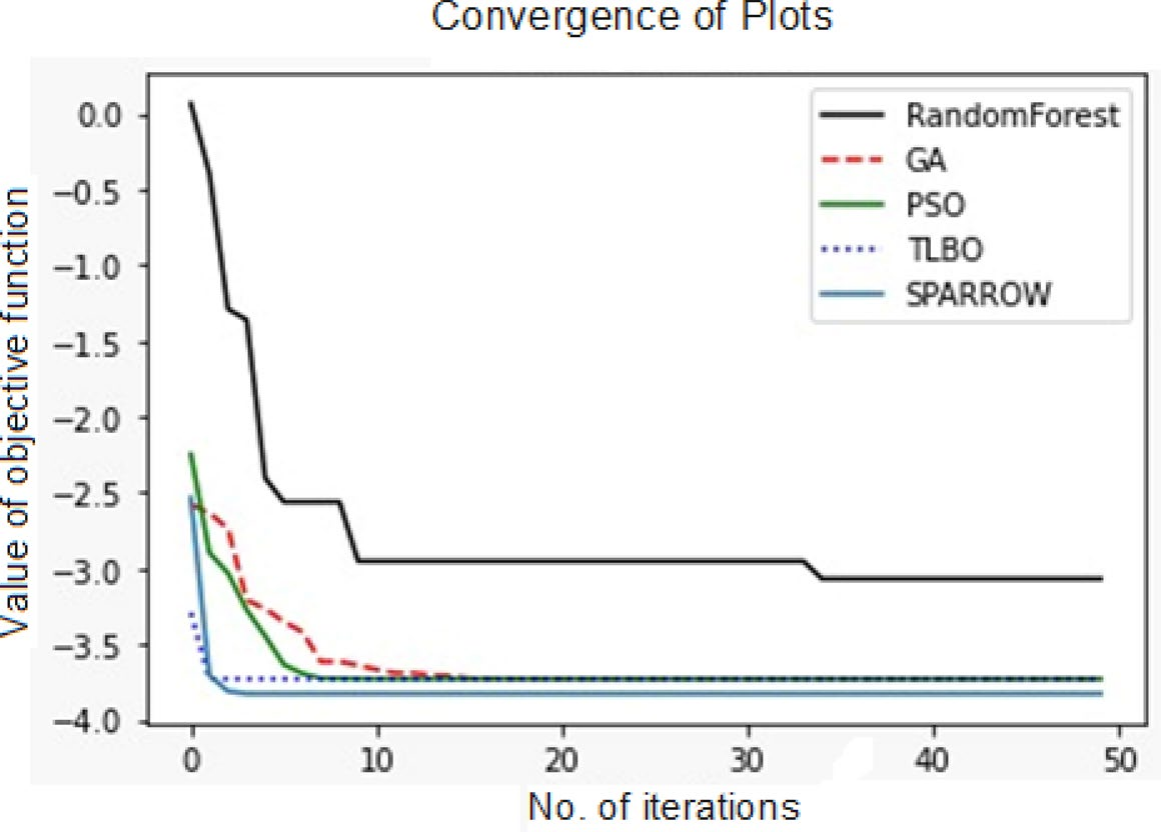
Convergence of Metaheuristic Algorithms.

Figure 16 depicts that the RFA demonstrates unique characteristics in comparison to GA, PSO, TLBO and SSA. Particularly, RFA exhibits a distinct convergence pattern, achieving convergence relatively later than the aforementioned algorithms due to its inherent structure, which does not involve an explicit search process. Instead, RFA constructs decision trees based on random subsections of the available data and features. The differences between RFA and the other algorithms are multifaceted. Firstly, their nature and approach vary significantly. While RFA relies on ensemble learning through decision trees but, GA, PSO, TLBO and SSA employ evolutionary, swarm-based, teaching–learning, and sparrow-inspired mechanisms, respectively. Secondly, the search mechanisms employed differ; RFA’s approach focuses on constructing decision trees, while the others involve iterative exploration and exploitation of the solution space.

Moreover, the objective function optimization strategies diverge. While GA, PSO, TLBO, and SSA are explicitly designed for optimization tasks, RFA is primarily used for classification and regression purposes. Additionally, the parallelism capabilities of these algorithms vary. GA, PSO, TLBO, and SSA can be effectively parallelized due to their concurrent evaluation and evolution of multiple candidate solutions, whereas RFA’s parallelization is limited to some extent, primarily due to the independence of decision tree construction.

Nonetheless, the study results suggest that enhancing RFA’s performance may be achievable by increasing the number of iterations, thereby facilitating better convergence and yielding superior outcomes. Based on Table 5, RFA demonstrated superior performance in achieving higher MR and lower TW compared to other nature-inspired metaheuristic algorithms.

**Table 5:** Optimal values from the various Metaheuristic Algorithms.

Algorithm	Responses predicted based on input parameters					% of average deviation from experimental results
	Applied voltage (V)	Elect.con. (wt%)	Nitrogen gas flow rate (L/min)	MR (mg)	TW (mg)	
GA	119.63	14.95	4.88	2.506	− 0.885	8.11
PSO	119.98	15.44	4.90	2.59	− 0.845	7.02
TLBO	119.41	12.506	4.59	2.054	− 0.985	7.13
SSA	119.06	11.077	3.63	1.213	− 0.431	7.75
RFA	119.89	20.107	4.31	3.123	− 0.486	8.21

Although RFA is primarily an ensemble learning model rather than an optimization technique, its predicted parameter values were very close to those obtained by metaheuristic algorithms, providing strong evidence of its robustness and consistency. Experimental results confirmed that at 120 V, 20 wt% electrolyte concentration, and 4 L/min nitrogen flow rate, the process achieved MR above 3 mg with TW below − 0.5 mg, in line with initial conclusions. The deviation between RFA predictions and experimental values was ~ 8%, further validating the reliability of the model.

**Fig. 17:**
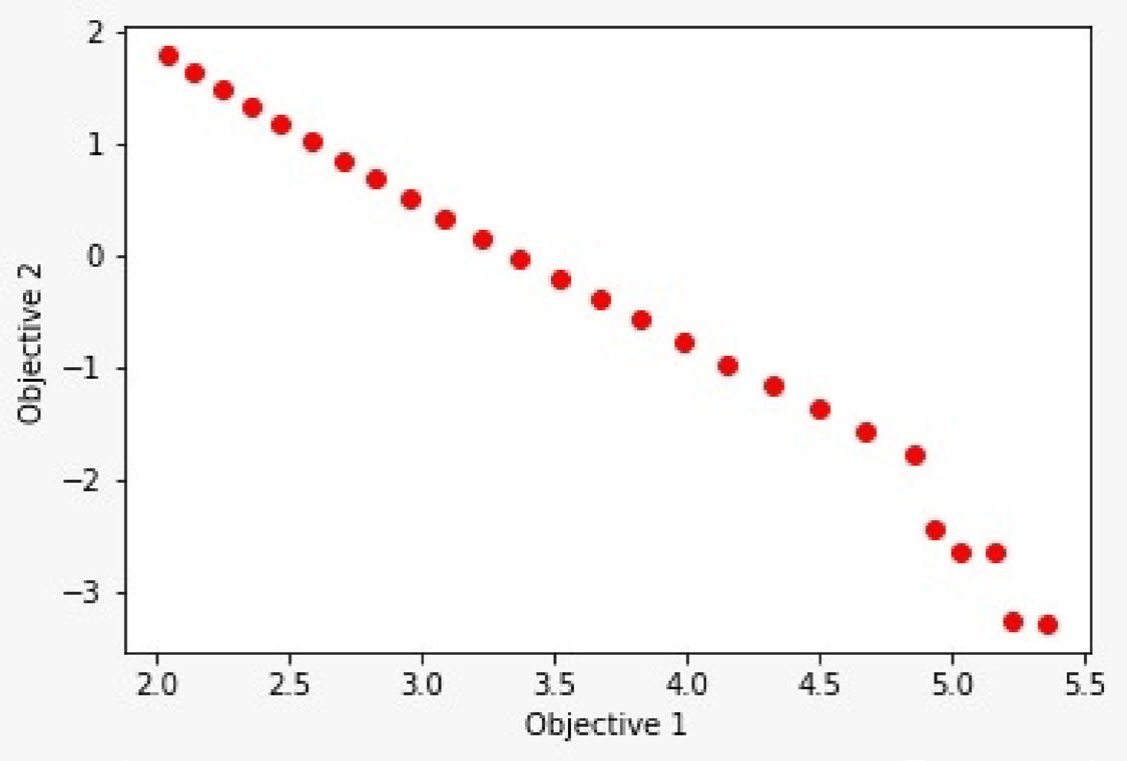
Pareto front for RFA.

Moreover, Fig. 17 presents a Pareto front, highlighting the trade-offs between objectives and identifying feasible solution sets.

Each red dot represents a non-dominated solution, meaning no other solution is better in both objectives simultaneously. This Pareto front indicates the optimal balance between removing more material efficiently and minimizing tool degradation. In multi-objective optimization, especially in industrial applications, not all solutions can simultaneously optimize all outcomes. This helps decision-makers choose the best compromise based on their priorities. Borosilicate glass has found widespread use in large-scale applications within MEMS and NEMS, serving as substrates for crystal growth and in micro-pumps. Thus, the Random Forest model facilitated the identification of key influencing factors and enabled reliable prediction of ECDµM performance with respect to enhanced MR and minimized TW.

However, this study was limited to borosilicate glass specimens of a single size and thickness, processed using a fixed electrode configuration and a defined range of NaOH electrolyte concentrations. Only three process parameters—applied voltage, electrolyte concentration, and nitrogen gas flow rate—were considered, while other influencing factors such as duty cycle, pulse frequency and tool geometry were held constant. The Random Forest model was developed using the available experimental dataset, and its predictive capability remains constrained by the size and diversity of the data.

## Conclusions

This study demonstrates the effectiveness of nitrogen gas assisted ECDµM in improving machining performance and sustainability during micro-cavity production in borosilicate glass. The integration of nitrogen gas altered the discharge environment, leading to improved process stability, enhanced MR and reduced TW. The combined experimental, statistical, and data-driven analyses provide clear evidence of the benefits of nitrogen gas assistance under controlled operating conditions. The key findings are summarized below:

- Nitrogen gas improved discharge stability and thermal control, resulting in increased MR and reduced TW across the investigated parameter range.
- At 120 V and 30 wt% NaOH concentration, increasing the nitrogen gas flow rate from 3 L/min to 5 L/min maintained MR at approximately 4 mg while reducing TW from 3 to 2 mg, indicating improved process stability without loss of productivity.
- The reduction in TW corresponds to an estimated 33% decrease in TW–related energy consumption, reflecting improved energy efficiency and lower demand for tool regeneration.
- Optimal performance was obtained at 134 V, 20 wt% NaOH, and 4 L/min nitrogen gas flow rate, yielding 5 mg MR with negative TW (− 1 mg), demonstrating the suitability of nitrogen gas assisted ECDµM for machining borosilicate glass materials.
- Compared with higher electrolyte concentrations, the optimal condition represents a 33% reduction in NaOH usage, highlighting the potential for reduced chemical consumption, lower hazardous waste generation and diminished environmental impact.
- GRA identified operating conditions consistent with experimental trends, while the Random Forest model predicted near-optimal parameters (119.89 V, 20.107 wt% NaOH, and 4.31 L/min nitrogen gas flow rate) with predicted outcomes of 3.123 mg MR and − 0.486 mg TW, showing an average deviation of approximately 8%.
- The close agreement between experimental results, statistical optimization, machine-learning predictions, and metaheuristic algorithms confirms the robustness and reliability of the proposed prediction framework.

Overall, the findings establish nitrogen gas assisted ECDµM as a stable, energy-efficient and environmentally responsible micromachining approach for producing repeatable, crack-free micro-features in borosilicate glass, with practical relevance for both laboratory-scale and industrial applications, including MEMS and NEMS.

Future work should focus on different glass materials and thicknesses, alternative electrolytes or mixed gaseous dielectric environments and an expanded set of process parameters. Advanced diagnostic techniques, such as high-speed imaging and in situ monitoring, will enable deeper insight into gas-film dynamics and discharge behavior. Further integration of machine learning with multi-objective optimization and validation under industrial operating conditions will strengthen the robustness and practical relevance of the proposed framework.

## Data Availability

The datasets used and/or analyzed during the current study available from the corresponding author on reasonable request.
