# Author Correction: Performance enhancement of electrochemical discharge micromachining of borosilicate glass using nitrogen gas assistance

**DOI:** 10.1038/s41598-026-57797-4

**Published:** 2026-06-16

**Authors:** Sekar Tamilperuvalathan, Vinoth Varadharaju, Sakthivel Rajamohan, Dhinesh Balasubramanian, Utku Kale, Artūras Kilikevičius

**Affiliations:** 1 Department of Mechanical Engineering, Government College of Technology, Coimbatore, India; 2 Department of Mechanical Engineering, Dhirajlal Gandhi College of Technology, Salem, India; 3 Department of Port Engineering, Lithuanian Maritime Academy (LMA) Vilnius Gediminas Technical University, Klaipėda, Lithuania; 4 Department of Aeronautics and Naval Architecture, Faculty of Transportation Engineering and Vehicle Engineering, Budapest University of Technology and Economics, Műegyetem Rkp. 3., Budapest, 1111 Hungary; 5 Mechanical Science Institute, Vilnius Gediminas Technical University, Plytinės G. 25, 10105 Vilnius, Lithuania

Correction to: *Scientific Reports* 10.1038/s41598-026-36060-w, published online 12 February 2026

The original version of this Article contained errors in the Reference list.

The incorrect and correct information is listed below.

Incorrect title:

- Reference 2: Pawar, P., Ballav, R. & Kumar, A. Micromachining of borosilicate glass: a state-of-the-art review. Mater. Today Proc. 4, 2813–2821. 10.1016/j.matpr.2017.02.163 (2017).
- Reference 5: Kumar, N., Mandal, N. & Das, A. K. Micro-electrochemical sparks machining: fabrication of microcomponents from nonconductive materials. In Micro Electro-fabrication, 277–315 (Elsevier, 2021).
- Reference 6: Liu, H. & Xieeryazidan, A. Gas-film characteristics and discharge energy distribution in ECDM. Micromachines 14, 1079. 10.3390/mi14061079 (2023).
- Reference 7: Wüthrich, R. et al. Spark-assisted chemical engraving in microfactory applications. J. Micromech. Microeng. 15, S276–S283. 10.1088/0960-1317/15/10/021 (2005).
- Reference 10: Bhargav, K. V. J. et al. Multi-response optimization of μ-ECDM of PMMA. CIRP J. Manuf. Sci. Technol. 38, 473–490. 10.1016/j.cirpj.2022.06.001 (2022).
- Reference 11: Sana, M. et al. Multi-response optimization of wire EDM of hardened AISI D2 and DC53 tool steels using Taguchi L18 and weighted S/N approach. Int. J. Adv. Manuf. Technol. 10.1007/s00170-025-16808-w (2025).
- Reference 19: Hassan, S. et al. Multi-objective optimization of complex feature machining on tool steels. J. Mater. Eng. Perform. 33, 12,109–12,123. 10.1007/s11665-024-09213-6 (2024).
- Reference 20: Ali, M. A. et al. GA-ANN optimization of EDM for hybrid aluminium composites. J. Mater. Res. Technol. 31, 4113–4127. https://d oi.org/10.1016/j.jmrt.2024.05.043 (2024).
- Reference 49: Manoharan, V. et al. Nitrogen gas-assisted ECDμM of glass using black widow optimization. Mater. Today Commun. 38, 108295. 10.1016/j.mtcomm.2024.108295 (2024).
- Reference 66: Wu, D., Jennings, C., Terpenny, J., Gao, R. X. & Kumara, S. A comparative study on machine learning algorithms for tool wear prediction in smart manufacturing. J. Manuf. Sci. Eng. 139, 071018. 10.1115/1.4036350 (2017).
- Reference 71: Suresh, S. et al. Sustainable friction stir spot welding of AA6061-T6 using an improved NS-TLBO algorithm. J. Mater. Res. Technol. 9, 11650–11674. 10.1016/j.jmrt.2020.08.086 (2020).

Correct title:

- Reference 2: Pawar, P., Ballav, R. & Kumar, A. Micromachining of borosilicate glass: A state of art review. Mater. Today Proc. 4, 2813–2821. 10.1016/j.matpr.2017.02.161 (2017).
- Reference 5: Manoharan, V. & Tamilperuvalathan, S. Prediction on enhanced electrochemical discharge machining behavior using hybrid deep neural network-based spotted hyena optimization. Int. J. Energy Res. 46, 9221–9241. 10.1002/er.7797 (2022).
- Reference 6: Liu, H. & Xieeryazidan, A. Study of gas film characteristics in electrochemical discharge machining and their effects on discharge energy distribution. Micromachines 14, 1079. 10.3390/mi14051079 (2023).
- Reference 7: Wüthrich, R., Fujisaki, K., Couthy, P., Hof, L. A. & Bleuler, H. Spark assisted chemical engraving (SACE) in microfactory. J. Micromech. Microeng. 15, S276–S280. 10.1088/0960-1317/15/10/S04 (2005).
- Reference 10: Bhargav, K. V. J., Balaji, P. S., Sahu, R. K. & Katiyar, J. K. Multi-response optimization and effect of tool rotation on micromachining of PMMA using an in-house developed μ-ECDM system. CIRP J. Manuf. Sci. Technol. 38, 473–490. 10.1016/j.cirpj.2022.05.020 (2022).
- Reference 11: Sana, M., Asad, M., Khan, A. et al. Using weighted signal-to-noise metric to tailor intricate geometries on heat treated AISI D2 and DC53 steel materials and optimizing for reduced spark gap formation. Int. J. Adv. Manuf. Technol. 141, 3759–3779. 10.1007/s00170-025-16808-w (2025).
- Reference 19: Hassan S., Asad M., Sana M. et al. Parametric analysis and multi-objective optimization for machining complex features on D2 and DC53 steels for tooling applications. J. Mater. Eng. Perform. 33, 12109–12123. https://link.springer.com/article/10.1007/s11665-024-09828-2 (2024).
- Reference 20: Ali M.A., Mufti N.A., Sana M., Tlija M., Farooq M.U. & Haber R. Enhancing high-speed EDM performance of hybrid aluminium matrix composite by genetic algorithm integrated neural network optimization. J. Mater. Res. Technol. 31, 4113–4127. 10.1016/j.jmrt.2024.07.077 (2024).
- Reference 49: Vijay Manoharan, Sekar Tamilperuvalathan, Prasanth Ponnusamy & Elango Natarajan. Exploring nitrogen gas-assisted ECDµM of glass by modified blackwidow optimization. Mater. Today Commun. 38, 108295. 10.1016/j.mtcomm.2024.108295 (2024).
- Reference 66: Wu, D., Jennings, C., Terpenny, J., Gao, R. X. & Kumara, S. A comparative study on machine learning algorithms for smart manufacturing: Tool wear prediction using random forests. J. Manuf. Sci. Eng. 139, 071018. 10.1115/1.4036350 (2017).
- Reference 71: Tiwari, A. & Pradhan, M. K. Applications of TLBO algorithm on various manufacturing processes: A review. Mater. Today Proc. 4, 1644–1652. 10.1016/j.matpr.2017.02.003 (2017).

Incorrect URL

- Reference 3: Kumar, M., Vaishya, R. O., Suri, N. M. & Gupta, A. Parametric optimization of travelling wire electrochemical discharge machining of borosilicate glass. Mater. Today Proc. 64, 1206–1210. 10.1016/j.matpr.2022.05.389 (2022).
- Reference 8: Kolhekar, K. R. & Sundaram, M. Gas-film characterization and its effect in ECDM. Precis. Eng. 53, 203–211. 10.1016/j.precisioneng.2018.03.012 (2018).
- Reference 9: Grover, S., Singh, S. & Mangal, S. K. Hybridization of μ-ECDM processes: a review. Mater. Today Proc. 80, 499–508. 10.1016/j.matpr.2023.03.221 (2023).
- Reference 14: Hannan, A. et al. Machining performance, economic and environmental analysis of EDM for SS310. Sci. Rep. 14, 1–21. 10.1038/s41598-024-68251-3 (2024).
- Reference 22: Sana, M. et al. Sustainable EDM using alumina-mixed dielectric. J. Clean. Prod. 441, 140926. 10.1016/j.jclepro.2024.140926 (2024).
- Reference 31: Ladeesh, V. G. & Manu, R. Performance evaluation and multi-response optimization of grinding-aided electrochemical discharge drilling (G-ECDD) of borosilicate glass. J. Braz.
- Reference 32: Bellubbi, S., Naik, R. & Sathisha, N. An experimental study of process parameters on material removal rate in ECDM process. Mater. Today Proc. 35, 298–302. 10.1016/j.matpr.2020.06.191 (2021).
- Reference 35: Antil, P., Singh, S. & Manna, A. Electrochemical discharge drilling of SiC reinforced polymer matrix composite using Taguchi’s grey relational analysis. Arab. J. Sci. Eng. 43, 1257–1266. 10.1007/s13369-017-2827-3 (2018).
- Reference 36: Tripathy, S. & Tripathy, D. K. Multi-attribute optimization of machining process parameters in powder-mixed electro-discharge machining using TOPSIS and grey relational analysis. Eng. Sci. Technol. Int. J. 19, 62–70. 10.1016/j.jestch.2015.06.003 (2016).
- Reference 37: Kumar, G., Sen, B., Ghosh, S. & Rao, P. V. Strategic enhancement of machinability in nickel-based superalloy using eco-benign hybrid nano-MQL approach. J. Manuf. Process. 127, 457–476. 10.1016/j.jmapro.2024.01.031 (2024).
- Reference 38: Kumar, J. et al. Experimental evaluation onto the machinability of sustainable Al/SiC/Gr hybrid composite using grey relational analysis. Mater. Today Proc. 68, A7–A15. 10.1016/j.matpr.2022.05.065 (2022).
- Reference 42: Shanmugasundar, G. et al. A comparative study of linear, random forest and AdaBoost regressions for modeling non-traditional machining. Processes 9, 2015. 10.3390/pr9122015 (2021).
- Reference 50: Charak, A. & Jawalkar, C. S. Experimental studies on micro-channeling of borosilicate glass using response surface methodology. SILICON 12, 1707–1721. 10.1007/s12633-019-00288-3 (2020).
- Reference 51: Cheng, C. P. et al. Study of gas film quality in electrochemical discharge machining. Int. J. Mach. Tools Manuf. 50, 689–697. 10.1016/j.ijmachtools.2010.04.002 (2010).
- Reference 52: Wüthrich, R. & Hof, L. A. The gas film in spark assisted chemical engraving (SACE)—A key element for micromachining applications. Int. J. Mach. Tools Manuf. 46, 828–835. 10.1016/j.ijmachtools.2005.09.014 (2006).
- Reference 53: Wei, C., Xu, K., Ni, J., Brzezinski, A. J. & Hu, D. A finite element-based model for electrochemical discharge machining in the discharge regime. Int. J. Adv. Manuf. Technol. 54, 987–995. 10.1007/s00170-010-2983-6 (2011).
- Reference 54: Zare Chavoshi, S. & Behagh, A. M. Influential control parameters in drilling hard-to-machine steel by electrochemical discharge machining. Int. J. Adv. Manuf. Technol. 71, 1883–1887. 10.1007/s00170-013-5580-0 (2014).
- Reference 55: Kulkarni, A., Sharan, R. & Lal, G. K. Experimental study of discharge mechanisms in electrochemical discharge machining. Int. J. Mach. Tools Manuf. 42, 1121–1127. 10.1016/S0890-6955(02)00038-0 (2002).
- Reference 56: Sen, B. et al. Exploring cryo-MQL medium for hard machining of Hastelloy C276: A multi-objective optimization approach. Int. J. Interact. Des. Manuf. 19, 4773–4786. 10.1007/s12008-024-01638-1 (2025).
- Reference 60: Ghosh, A. Electrochemical discharge machining: principle and possibilities. Int. J. Mach. Tools Manuf. 37, 435–447. 10.1016/S0890-6955(96)00025-4 (1997).
- Reference 62: Jain, V. K. et al. Analysis of electrochemical spark machining. Int. J. Mach. Tools Manuf. 39, 165–186. 10.1016/S0890-6955(98)00037-1 (1999).
- Reference 64: Sen, B. et al. Technoeconomic and environmental analysis of cryogenic- and MQL-assisted machining of Hastelloy X. Sci. Rep. 15, 1–16. 10.1038/s41598-025-00094-8 (2025).
- Reference 70: Singh, M. & Singh, S. Multi-objective optimization of EDM of Nimonic 75 using teaching–learning-based optimization. Mater. Today Proc. 24, 576–584. 10.1016/j.matpr.2020.04.340 (2020).

Correct URL:

- Reference 3: Kumar, M., Vaishya, R. O., Suri, N. M. & Gupta, A. Parametric optimization of traveling wire electrochemical discharge machining (TW-ECDM) process for aspect ratio during machining of borosilicate glass. Mater. Today Proc. 64, 1206–1210. 10.1016/j.matpr.2022.03.599 (2022).
- Reference 8: Kolhekar, K. R. & Sundaram, M. Study of gas film characterization and its effect in electrochemical discharge machining. Precis. Eng. 53, 203–211. 10.1016/j.precisioneng.2018.04.002 (2018).
- Reference 9: Grover, S., Singh, S. & Mangal, S. K. Hybridization of μ-electrochemical discharge machining (μ-ECDM) process: A review. Mater. Today Proc. 80, 499–508. 10.1016/j.matpr.2022.10.204 (2023).
- Reference 14: Hannan, A., Mehmood, S., Ali, M.A. et al. Machining performance, economic and environmental analyses and multi-criteria optimization of electric discharge machining for SS310 alloy. Sci Rep 14, 28930. 10.1038/s41598-024-79338-7 (2024).
- Reference 22: Sana M., Asad M., Farooq M.U., Anwar S. & Talha M. Sustainable electric discharge machining using alumina-mixed deionized water as dielectric: process modelling by artificial neural networks underpinning net-zero from industry. J. Clean. Prod. 441, 140926. 10.1016/j.jclepro.2024.140926 (2024).
- Reference 31: Ladeesh, V. G. & Manu, R. Performance evaluation and multi-response optimization of grinding-aided electrochemical discharge drilling (G-ECDD) of borosilicate glass. J. Braz. Soc. Mech. Sci. Eng. 40, 568. 10.1007/s40430-018-1489-6 (2018).
- Reference 32: Bellubbi, S., Naik, R. & Sathisha, N. An experimental study of process parameters on material removal rate in ECDM process. Mater. Today Proc. 35, 298–302. 10.1016/j.matpr.2020.01.510 (2021).
- Reference 35: Antil, P., Singh, S. & Manna, A. Electrochemical discharge drilling of SiC reinforced polymer matrix composite using Taguchi’s grey relational analysis. Arab. J. Sci. Eng. 43, 1257–1266 10.1007/s13369-017-2822-6 (2018).
- Reference 36: Tripathy, S. & Tripathy, D. K. Multi-attribute optimization of machining process parameters in powder mixed electro-discharge machining using TOPSIS and grey relational analysis. Eng. Sci. Technol. Int. J. 19, 62–70. 10.1016/j.jestch.2015.07.010 (2016).
- Reference 37: Kumar, G., Sen, B., Ghosh, S. & Rao, P. V. Strategic enhancement of machinability in nickel-based superalloy using eco-benign hybrid nano-MQL approach. J. Manuf. Process. 127, 457–476. 10.1016/j.jmapro.2024.08.015 (2024).
- Reference 38: Kumar, J., Gagandeep, Sharma, S., Chohan, J., Kumar, R., Singh, S., Obaid, A. J. & Joshi, A. Experimental evaluation onto the machinability of sustainable Al/SiC/Gr hybrid composite using grey relational analysis. Mater. Today Proc. 68, A7–A15. 10.1016/j.matpr.2022.11.098 (2022).
- Reference 42: Shanmugasundar, G., Vanitha, M., Čep, R., Kumar, V., Kalita, K. & Ramachandran, M. A comparative study of linear, random forest and AdaBoost regressions for modeling non-traditional machining. Processes 9, 2015. 10.3390/pr9112015 (2021).
- Reference 50: Charak, A. & Jawalkar, C. S. Experimental studies in micro channelling on borosilicate glass using RSM optimization technique. Silicon 12, 1707–1721. 10.1007/s12633-019-00269-4 (2020).
- Reference 51: Cheng, C.-P., Wu, K.-L., Mai, C.-C., Yang, C.-K., Hsu, Y.-S. & Yan, B.-H. Study of gas film quality in electrochemical discharge machining. Int. J. Mach. Tools Manuf. 50, 689–697. 10.1016/j.ijmachtools.2010.04.012 (2010).
- Reference 52: Wüthrich, R. & Hof, L. A. The gas film in spark assisted chemical engraving (SACE)—a key element for micro-machining applications. Int. J. Mach. Tools Manuf. 46, 828–835. 10.1016/j.ijmachtools.2005.07.029 (2006).
- Reference 53: Wei, C., Xu, K., Ni, J., Brzezinski, A. J. & Hu, D. A finite element-based model for electrochemical discharge machining in the discharge regime. Int. J. Adv. Manuf. Technol. 54, 987–995. 10.1007/s00170-010-3000-0 (2011).
- Reference 54: Zare Chavoshi, S. & Behagh, A. M. A note on influential control parameters for drilling of hard-to-machine steel by electrochemical discharge machining. Int. J. Adv. Manuf. Technol. 71, 1883–1887. 10.1007/s00170-014-5646-5 (2014).
- Reference 55: Kulkarni, A., Sharan, R. & Lal, G. K. An experimental study of discharge mechanism in electrochemical discharge machining. Int. J. Mach. Tools Manuf. 42, 1121–1127. 10.1016/S0890-6955(02)00058-5 (2002).
- Reference 56: Sen, B., Bhowmik, A., Rachchh, N. et al. Exploring cryo-MQL medium for hard machining of Hastelloy C276: a multi-objective optimization approach. Int. J. Interact. Des. Manuf. 19, 4773–4786. 10.1007/s12008-024-02069-6 (2025).
- Reference 60: Ghosh, A. Electrochemical discharge machining: Principle and possibilities. Sadhana 22, 435–447. 10.1007/BF02744482 (1997).
- Reference 62: Jain, V. K., Dixit, P. M. & Pandey, P. M. On the analysis of the electrochemical spark machining process. Int. J. Mach. Tools Manuf. 39, 165–186. 10.1016/S0890-6955(98)00010-8 (1999).
- Reference 64: Sen, B., Murali Krishnam Raju, V. V., Kumar, R. et al. Technoeconomic and environmental analysis of cryogenic and MQL-assisted machining of Hastelloy X. Sci. Rep. 15, 21816. 10.1038/s41598-025-07526-0 (2025).
- Reference 70: Singh, M. & Singh, S. Multi-objective optimization of electrical discharge machining of Nimonic 75 using teaching learning based optimization (TLBO) algorithm. Mater. Today Proc. 24, 576–584. 10.1016/j.matpr.2020.04.311 (2020).

Missing URL

- Reference 4: Kumar, N., Mandal, N. & Das, A. K. Micro-electrochemical sparks machining: fabrication of microcomponents from nonconductive materials. In Micro Electro-fabrication, 277–315 (Elsevier, 2021).
- Reference 33: Sen, B., Bhowmik, A., Prakash, C. & Ammarullah, M. I. Prediction of specific cutting energy consumption in eco-benign lubricating environment for biomedical industry applications: Exploring efficacy of GEP, ANN, and RSM models. AIP Adv. 10.1063/5.0XXXXX (2024).
- Reference 40: Bhanumathy, S. et al. Prediction of material removal rate of SS304 alloy in electrochemical micromachining using machine learning. Mater. Today Proc. 10.1016/j.matpr.2023.02.XXX (2023).
- Reference 44: Sen, B. et al. Alumina-enriched sunflower bio-oil in machining of Hastelloy C-276: A fuzzy Mamdani model-aided sustainable manufacturing paradigm. Sci. Rep. 14, 1–24. 10.1038/s41598-024-XXXXX (2024).
- Reference 45: Sen, B. et al. Minimum quantity blended bio-lubricants for sustainable machining of superalloy: An MCDM model-based study. AIP Adv. 10.1063/5.0XXXXX (2024).

Updated URL

- Reference 4: Kumar, N., Mandal, N. & Das, A. K. Micro-electrochemical sparks machining: fabrication of microcomponents from nonconductive materials. In Micro Electro-fabrication, 277–315 10.1016/B978-0-12-820049-0.00012-8 (2021).
- Reference 33: Sen, B., Bhowmik, A., Prakash, C. & Ammarullah, M. I. Prediction of specific cutting energy consumption in eco-benign lubricating environment for biomedical industry applications: Exploring efficacy of GEP, ANN, and RSM models. AIP Adv. 14(8), 085216. 10.1063/5.0217508 (2024).
- Reference 40: Bhanumathy, S., Prasanth, B. M., Anitha, B., Mohanakanna, R., Nandhakumar, E. & Hariharan, P. Prediction of material removal rate of SS304 alloy in electrochemical micromachining using machine learning. Mater. Today Proc. 10.1016/j.matpr.2023.07.234 (2023).
- Reference 44: Sen, B., Bhowmik, A., Singh, G. et al. Alumina-enriched sunflower bio-oil in machining of Hastelloy C-276: a fuzzy Mamdani model-aided sustainable manufacturing paradigm. Sci. Rep. 14, 29194. 10.1038/s41598-024-80254-z (2024).
- Reference 45: Sen, B., Kothapalli, S. K., Kumar, R., Manjunath, C., Abdullah, I., Singh, G. & Santhosh, A. J. Minimum quantity blended bio-lubricants for sustainable machining of superalloy: An MCDM model-based study. AIP Adv. 14(7), 075224. 10.1063/5.0222561 (2024).

Incorrect authors and URL

- Reference 12: Singh Walia, A. et al. Producing micro-impressions on Al6061 using alumina-mixed dielectric in EDM. J. Micromech. Microeng. 35, 035011. 10.1088/1361-6439/ad0f3b (2025).

Correct authors and URL

- Asad M., Sana M., Farooq M.U. & Tlija M. Producing micro impressions on Al6061 under alumina-mixed deionized water as dielectric during electric discharge machining. J. Micromech. Microeng. 35, 035011. https://iopscience.iop.org/article/10.1088/1361-6439/adb044 (2025).

Incorrect title and URL

- Reference 13: Sana, M. et al. Sustainability-targeted optimization of EDM using neural networks. Sci. Rep. 15, 1–30. 10.1038/s41598-025-00000-x (2025).
- Reference 15: Hurairah, M. A. et al. GA-optimized ANN modelling for graphene-mixed dielectric EDM. Arab. J. Sci. Eng. 49, 15649–15666. 10.1007/s13369-024-08512-3 (2024).
- Reference 16: Tlija, M. et al. EDM optimization of AISI D2 steel for biomedical tooling. AIP Adv. 14, 105129. 10.1063/5.0203124 (2024).
- Reference 17: Sana, M. et al. ANN-based modelling of cryogenic and nano-dielectric EDM. J. Braz. Soc. Mech. Sci. Eng. 46, 1–19. 10.1007/s40430-024-04732-y (2024).
- Reference 18: Farooq, M. U. et al. Process optimization of micro-complex geometry machining of Ti-6Al-4V. Int. J. Interact. Des. Manuf. 18, 4573–4593. 10.1007/s12008-024-01492-1 (2024).
- Reference 21: Farooq, M. U. & Anwar, S. Reducing micro-machining errors in EDM using non-ionic liquids. Mater. Manuf. Process. 39, 449–464. 10.1080/10426914.2023.2279441 (2024).
- Reference 23: Farooq, M. U., Anwar, S., Ali, M. A., Hassan, A. & Mushtaq, R. T. Exploring a wide parametric range for tool electrode selection based on surface characterization and machining rate in powder-mixed electric discharge machining of Ti6Al4V ELI. Int. J. Adv. Manuf. Technol. 129, 2823–2841. 10.1007/s00170-023-11542-9 (2023).
- Reference 24: Farooq, M. U. et al. Electric discharge machining of Ti6Al4V ELI for biomedical applications: Parametric analysis of surface functionalization and tribological characterization. Materials 16, 4458. 10.3390/ma16134458 (2023).
- Reference 46: Kumaravel, P. et al. Improvement of μ-ECDM using N2-assisted NaOH electrolyte. Int. J. Electrochem. Sci. 17, 220747. 10.20964/2022.07.47 (2022).
- Reference 50: Charak, A. & Jawalkar, C. S. Experimental studies on micro-channeling of borosilicate glass using response surface methodology. SILICON 12, 1707–1721. 10.1007/s12633-019-00288-3 (2020).
- Reference 65: Sen, B., Dasgupta, A. & Bhowmik, A. Optimizing wire-cut EDM parameters through evolutionary algorithm: a study for improving cost efficiency in turbo-machinery manufacturing. Int. J. Interact. Des. Manuf. 19, 2049–2060. 10.1007/s12008-024-02001-y (2025).
- Reference 67: Madushani, S., Sandamal, K. & Rangajeewa, T. A genetic algorithm-based decision framework for incorporating climate impact in pavement maintenance planning. Period. Polytech. Transp. Eng. 52, 120–127. 10.3311/PPtr.21365 (2024).
- Reference 69: Kunhare, N., Tiwari, R. & Dhar, J. Particle swarm optimization-based feature selection for intrusion detection systems. Sādhanā 45, 1–14. 10.1007/s12046-020-01383-0 (2020).
- Reference 72: Suresh, S. et al. Sustainable friction stir spot welding of AA6061-T6 using an improved NS-TLBO algorithm. J. Mater. Res. Technol. 9, 11650–11674. 10.1016/j.jmrt.2020.08.086 (2020).
- Reference 76: Sen, B. et al. Comparative analysis of NSGA-II and TLBO for sustainable machining of Inconel 690. J. Mater. Eng. Perform. 34, 17503–17518. 10.1007/s11665-024-09301-7 (2024).

Correct title and URL

- Reference 13: Sana, M., Asad, M., Farooq, M.U. et al. Sustainability metrics targeted optimization and electric discharge process modelling by neural networks. Sci Rep 15, 3375. 10.1038/s41598-024-78883-5 (2025).
- Reference 15: Hurairah M.A., Sana M., Farooq M.U. et al. Genetic algorithm-based optimization of artificial neural network of process parameters and characterization of machining errors in graphene mixed dielectric. Arab. J. Sci. Eng. 49, 15649–15666. 10.1007/s13369-024-09029-y (2024).
- Reference 16: Tlija M., Rashid T., Sana M., Farooq M.U., Ammarullah M.I. AISI D2 steel machining and manufacturing process optimization for tooling applications in biomedical industry. AIP Adv. 14, 105129. 10.1063/5.0217712 (2024).
- Reference 17: Sana M., Khan A., Farooq M.U. et al. Artificial neural networks-based modelling of effects of cryogenic electrode treatment, nano-powder, and surfactant-mixed dielectrics on wear performance and dimensional errors on superalloy machining. J. Braz. Soc. Mech. Sci. Eng. 46, 539. https://link.springer.com/article/10.1007/s40430-024-05100-9 (2024).
- Reference 18: Farooq M.U., Ali M.A., Anwar S. et al. Process parameters optimization and performance analysis of micro-complex geometry machining on Ti6Al4V. Int. J. Interact. Des. Manuf. 18, 4573–4593. https://link.springer.com/article/10.1007/s12008-023-01711-z (2024).
- Reference 21: Farooq M.U., Anwar S. & Hurairah A. Reducing micro-machining errors during electric discharge machining of titanium alloy using nonionic liquids. Mater. Manuf. Process. 39, 449–464. https://www.tandfonline.com/doi/full/10.1080/10426914.2023.2236199 (2024).
- Reference 23: Farooq M.U., Anwar S., Ali M.A. et al. Exploring wide-parametric range for tool electrode selection based on surface characterization and machining rate employing powder-mixed electric discharge machining process for Ti6Al4V ELI. Int. J. Adv. Manuf. Technol. 129, 2823–2841. https://link.springer.com/article/10.1007/s00170-023-12469-9 (2023).
- Reference 24: Farooq M.U., Anwar S., Bhatti H.A., Kumar M.S., Ali M.A. & Ammarullah M.I. Electric discharge machining of Ti6Al4V ELI in biomedical industry: parametric analysis of surface functionalization and tribological characterization. Materials 16, 4458. https://www.mdpi.com/1996-1944/16/12/4458 (2023).
- Reference 46: Kumaravel, P., Suresh, P., Raja, K. V. & Sekar, T. Improvement of micro-electrochemical discharge machining of austenitic stainless steel 316L using NaOH electrolyte containing N₂. Int. J. Electrochem. Sci. 17, 220747. 10.20964/2022.07.53 (2022).
- Reference 50: Charak, A. & Jawalkar, C. S. Experimental studies in micro channelling on borosilicate glass using RSM optimization technique. Silicon 12, 1707–1721. 10.1007/s12633-019-00269-4 (2020).
- Reference 65: Sen, B., Dasgupta, A. & Bhowmik, A. Optimizing wire-cut EDM parameters through evolutionary algorithm: a study for improving cost efficiency in turbo-machinery manufacturing. Int. J. Interact. Des. Manuf. 19, 2049–2060. 10.1007/s12008-024-02001-y (2025).
- Reference 67: Madushani, S., Sandamal, K. & Rangajeewa, T. A genetic algorithm-based decision framework to incorporate climate impact on pavement maintenance planning. Period. Polytech. Transp. Eng. 52, 120–127. 10.3311/PPtr.22290 (2024).
- Reference 69: Kunhare, N., Tiwari, R. & Dhar, J. Particle swarm optimization and feature selection for intrusion detection system. Sadhana 45, 109. 10.1007/s12046-020-1308-5 (2020).
- Reference 72: Suresh, N., Elango, N., Venkatesan, K., Lim, W. H., Palanikumar, K. & Rajesh, S. Sustainable friction stir spot welding of 6061-T6 aluminium alloy using improved non-dominated sorting teaching learning algorithm. J. Mater. Res. Technol. 9, 11650–11674. 10.1016/j.jmrt.2020.08.043 (2020).
- Reference 76: Sen, B., Kumar, R., Kanabar, B. et al. Comparative analysis of NSGA-II and TLBO for optimizing machining parameters of Inconel 690: A sustainable manufacturing paradigm. J. Mater. Eng. Perform. 34, 17503–17518. 10.1007/s11665-024-10539-x (2025).

Incorrect publication year and URL:

- Reference 40: Bhanumathy, S. et al. Prediction of material removal rate of SS304 alloy in electrochemical micromachining using machine learning. Mater. Today Proc. 10.1016/j.matpr.2023.02.XXX (2023).

Correct publication year and URL:

- Reference 40: Bhanumathy, S., Prasanth, B. M., Anitha, B., Mohanakanna, R., Nandhakumar, E. & Hariharan, P. Prediction of material removal rate of SS304 alloy in electrochemical micromachining using machine learning. Mater. Today Proc. 10.1016/j.matpr.2023.07.234 (2023).

Incorrect authors, title and URL

- Reference 30: Paul, L. & Hiremath, S. S. Response surface modelling of micro-holes in the electrochemical discharge machining process. Procedia Eng. 64, 1395–1404. 10.1016/j.proeng.2013.09.214 (2013).

Correct authors, title and URL

- Reference 30: Lijo, P. & Somashekhar S. Hiremath. Response surface modelling of micro holes in electrochemical discharge machining process. Procedia Engineering 64, 1395–1404. 10.1016/j.proeng.2013.09.221 (2013).

The original Article has been corrected.

